# Genetic Diversity of the Noncoding Control Region of the Novel Human Polyomaviruses

**DOI:** 10.3390/v12121406

**Published:** 2020-12-07

**Authors:** Ugo Moens, Carla Prezioso, Valeria Pietropaolo

**Affiliations:** 1 Department of Medical Biology, Faculty of Health Sciences, University of Tromsø—The Arctic University of Norway, 9037 Tromsø, Norway; 2 Department of Public Health and Infectious Diseases, “Sapienza” University of Rome, 00185 Rome, Italy; carla.prezioso@uniroma1.it (C.P.); valeria.pietropaolo@uniroma1.it (V.P.); 3 IRCSS San Raffaele Pisana, Microbiology of Chronic Neuro-degenerative Pathologies, 00160 Rome, Italy

**Keywords:** disease, Merkel cell carcinoma, mutation, NCCR, novel human polyomaviruses, transcription factor binding sites

## Abstract

The genomes of polyomaviruses are characterized by their tripartite organization with an early region, a late region and a noncoding control region (NCCR). The early region encodes proteins involved in replication and transcription of the viral genome, while expression of the late region generates the capsid proteins. Transcription regulatory sequences for expression of the early and late genes, as well as the origin of replication are encompassed in the NCCR. Cell tropism of polyomaviruses not only depends on the appropriate receptors on the host cell, but cell-specific expression of the viral genes is also governed by the NCCR. Thus far, 15 polyomaviruses have been isolated from humans, though it remains to be established whether all of them are genuine human polyomaviruses (HPyVs). The sequences of the NCCR of these HPyVs show high genetic variability and have been best studied in the human polyomaviruses BK and JC. Rearranged NCCRs in BKPyV and JCPyV, the first HPyVs to be discovered approximately 30 years ago, have been associated with the pathogenic properties of these viruses in nephropathy and progressive multifocal leukoencephalopathy, respectively. Since 2007, thirteen novel PyVs have been isolated from humans: KIPyV, WUPyV, MCPyV, HPyV6, HPyV7, TSPyV, HPyV9, HPyV10, STLPyV, HPyV12, NJPyV, LIPyV and QPyV. This review describes all NCCR variants of the new HPyVs that have been reported in the literature and discusses the possible consequences of NCCR diversity in terms of promoter strength, putative transcription factor binding sites and possible association with diseases.

## 1. Introduction: Human Polyomaviruses

Polyomaviruses (PyVs) are non-enveloped viruses that are typically 40–45 nm in diameter, and that possess a double-stranded circular genome of around 5000 base-pairs. Birds and mammals, including humans, are natural hosts for PyVs [1,2]. Recently, PyVs have also been isolated from fish [3,4]. So far, 15 different polyomaviruses have been isolated from human samples. The first human polyomaviruses, BKPyV and JCPyV, were identified in 1971 [5,6]. In 2007, two new human polyomaviruses (Karolinska Institute PyV (KIPyV) [7] and Washington University PyV (WUPyV) [8] were detected, and in the following years, Merkel cell PyV (MCPyV) [9], HPyV6 [10], HPyV7 [10], Trichodisplasia spinulosa PyV (TSPyV) [11], HPyV9 [12], HPyV10 [13], Saint Louis PyV (STLPyV) [14], HPyV12 [15], New Jersey PyV (NJPyV) [16], Lyon IARC PyV (LIPyV) [17], and Quebec PyV [18] have been described. Their original source of isolation and association with human diseases is summarized in Table 1.

Whether all of these are genuine human polyomaviruses (HPyVs) remains to be determined. BKPyV, JCPyV, KIPyV, WUPyV, MCPyV, HPyV6, HPyV, TSPyV, HPyV9, HPyV10, STLPyV, HPyV12, and NJPyV are classified as human polyomaviruses by the International Committee of Taxonomy of Viruses [19,20], LIPyV has only been very recently described, while LIPyV DNA was originally detected in human skin [17]. LIPyV seroreactivity in healthy individuals is ~5% in healthy individuals and much lower than the seroprevalence of the other HPyVs, which is between 50–100% [21]. Accordingly, LIPyV DNA was not detected or present in <2% of examined skin, eyebrow hair, gargle samples and tonsil samples [17,22,23]. Moreover, LIPyV DNA was frequently detected in the feces of cats [24], suggesting that it may be a feline PyV rather than a HPyV. QPyV DNA was detected in the feces of one patient [18], and the seroprevalence of this PyV has not been examined. Despite its original identification in human liver, gastro-intestinal tract and colon tissue and a VP1 seropositivity (respectively LT seropositivity) between ~20–90% (respectively 30–40%) in healthy adults or malignant and non-malignant gastro-intestinal tract patients [15,25], HPyV12 DNA could not be detected in numerous human samples from different sources [22,26,27,28,29,30]. Moreover, the group of Feltkamp reported that HPyV12 seroprevalence is only around 5% [21,31]. A nearly identical HPyV12 variant was isolated from shrew, suggesting that HPyV12 may be transmitted from shrew to humans, or that human HPyV12 positive samples were contaminated [32].

## 2. The Polyomavirus Genome: The Noncoding Control Region

**Figure 1:**
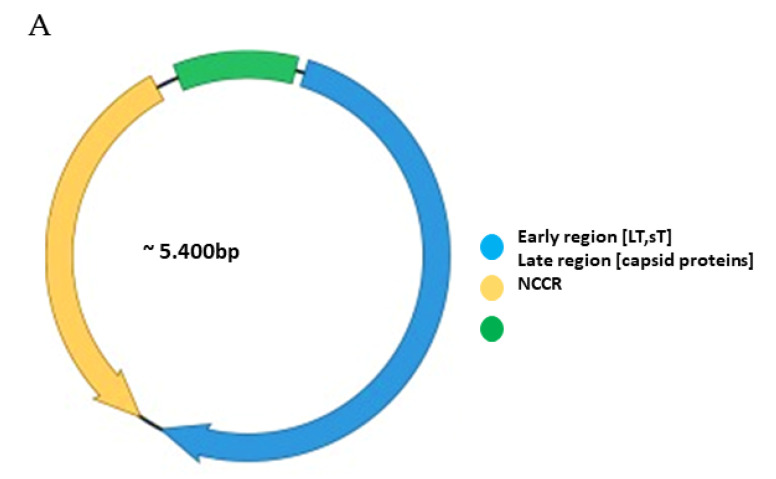

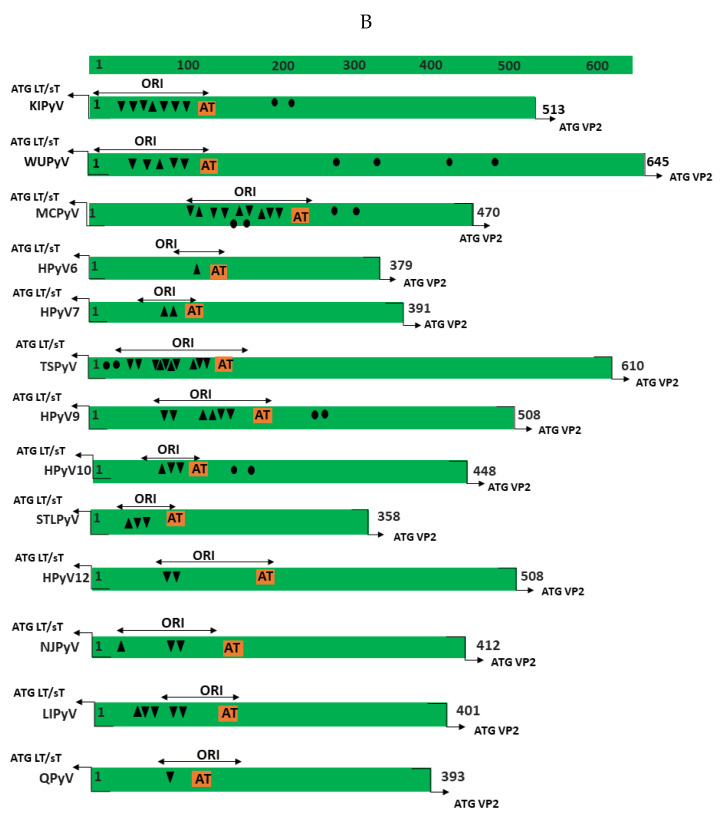

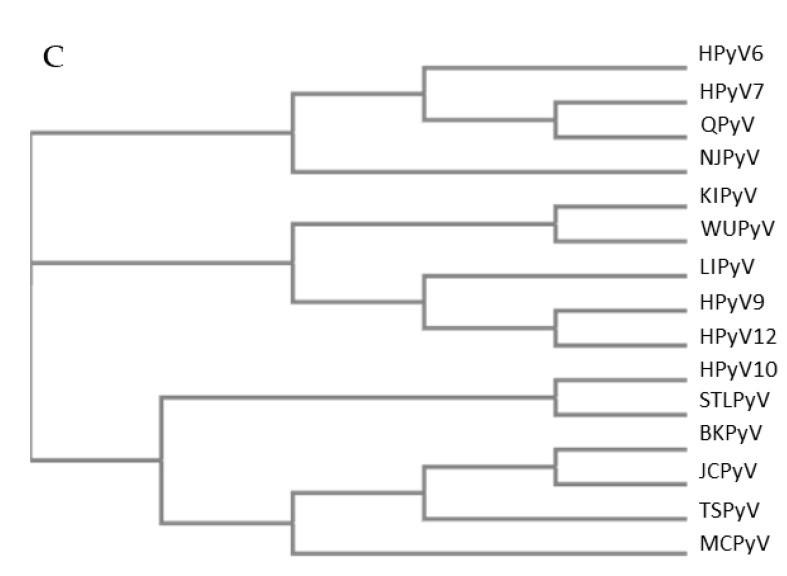
Genomic organization of the human polyomaviruses (HPyVs) genome and the structure of the noncoding control region (NCCR). (**A**) The circular dsDNA genome consists of the early and late regions that encode regulatory and structural proteins, respectively. Interspersed is the NCCR. (**B**) Schematic presentation of the NCCR of the novel HPyVs. The NCCR is the region between the start codon of Large T antigen (LT) and Small T antigen (sT) and the start codon of VP2. The AT-rich region (AT), repeated sequences (black dots), and LT binding motifs (upward pointing triangle = 5′-GRGGC-3′; downward pointing triangle = 5′-GCCYC-3′) are shown. (**C**) Phylogenetic tree bases on NCCR sequences of the different HPyVs. This is a neighbor-joining tree without distance corrections using Clustal Omega multiple sequence alignment [34].

Functionally, the PyV genome is tripartite consisting of the early region, the late region, and the noncoding control region (NCCR) (Figure 1A). The early region codes for regulatory proteins involved in replication and transcription of the viral genome. The major early proteins are large T-antigen (LT) and small t-antigen (sT). The late region codes for the structural proteins VP1, VP2 and VP3 that form the capsid. VP1 is the major capsid protein, while VP2 and VP3 are the minor capsid proteins [1,2]. However, not all PyVs express VP3 [33]. Interspersed between the early and late region are sequences that do not code for viral proteins, and is referred to as the NCCR.

Studies with simian virus 40 (SV40 or Macaca mulatta polyomavirus 1) and murine polyomaviruses have been pivotal in unveiling the functions of this region. The SV40 NCCR contains the origin of replication (ori), which consists of GRGGC motifs to which LT binds and is flanked by an AT-rich sequence and an easily denaturated imperfect palindrome [35,36]. Binding of LT to these motifs is also involved in regulation of viral transcription [37,38]. The NCCR also contains promoter and enhancer elements that control early and late transcription [39,40]. SV40 directly isolated from its natural host, rhesus monkey, has a NCCR that consists of an AT-rich region, triple GC-rich 21 base-pairs (bp) repeats, and a single 72 bp element. The 21 bp repeats contain the LT binding motif (GRGGC; [41,42]). This NCCR organization is known as the archetype. SV40 adapted to grow in cell culture has a duplication of this 72 bp element, with this type of NCCR referred to as prototype [43,44]. SV40 isolated from human tumors usually contain a single 72 bp repeat [43]. Rearrangements in the SV40 NCCR affect viral transcription and replication, as well as oncogenic properties of the virus [45,46]. The Mouse polyomavirus (Mus musculus polyomavirus 1; MPyV) NCCR encompasses the ori consisting of an AT-tract and a GC-rich (LT binding motifs) inverted repeat, and the transcription regulatory domains A (or α) and B (or β), C and D [47,48,49,50]. Alterations in the MPyV NCCR have an effect on viral replication in cell culture and in the host, the host range, and in vitro transformation [51,52,53,54,55].

The NCCR of the HPyVs varies between 267 bp (JCPyV CY-strain; accession number AB038249) to 645 bp (WUPyV prototype; accession number NC_009539) (see Figure S1 for the NCCR sequences of the novel HPyV), and similar to the NCCR of SV40 and MPyV, the NCCR of HPyVs also contain the origin of replication, LT binding motifs, and AT-rich region (Figure 1B). This region of the genome displays little or no sequence identity between the different HPyV species (Figure S2). A neighbor-joining tree without distance corrections shows which NCCRs are most closely related (Figure 1C).

The diversification based on the presence of a certain NCCR rearranged structure contributed to determining HPyVs strains as “archetype” or prototype”. The importance of the NCCR rearrangements during HPyVs infection became obvious when different strains of JCPyV were examined. The archetype JCPyV NCCR strain (CY) is divided into six boxes named A (36 bp), B (23 bp), C (55 bp), D (66 bp), E (18 bp), and F (69 bp) and contains the origin of replication (ORI), the promoter and the enhancer elements [56]. The NCCR harbored transcription factor binding sites such as the nuclear transcription factor-1 (NF1), a JCPyV cell-specific regulator of promoter and enhancer activity [57,58], the activating protein 1 (AP1), involved in JCPyV early gene expression [57,59], and the specificity protein-1 (SP1) able to regulate JCPyV transcription [57,60]. The archetype NCCR is considered the transmissible form of the virus among the population, and could be released into the urine of healthy individuals due to periodic and subclinical reactivation in the kidney [61,62]. In contrast, in the context of immunosuppression or during immunomodulatory therapy or in AIDS patients, JCPyV can reactivate from latency to cause a fatal pathology of the central nervous system (CNS), known as progressive multifocal leukoencephalopathy (PML) [61]. JCPyV variants carrying rearranged NCCR were usually isolated from PML patients. The prototype Mad-1 strain is the most studied variant of JCPyV and is characterized by 98-bp tandem repeats in the NCCR late proximal region (arranged as ORI-A-C-E-A-C-E-F), and is able to increase viral gene expression in human glial cells, thereby indicating that it is involved in controlling cell gene expression [63,64,65]. The enhancer repeats found in the Mad-1 strain are lacking in the archetype JCPyV strains isolated from the urine of healthy individuals [64]. Additional NCCR rearrangements are implicated in the development of the JCPyV pathogenic strains. In fact, in a significant proportion of JCPyV archetype isolates, short deletions or duplications were observed, corroborating that this region is highly unstable [66,67]. Therefore, it is possible to assume that subsequent archetypal NCRR rearrangements could determine the onset of PML strains, such as Mad-1 [68].

Based on the occurrence in the NCRR of transcriptional enhancer repeat elements, BKPyV isolates can also be identified as archetype and prototype strains. The archetype BKPyV WW strain, characterized by five blocks named O (35 bp), which includes the origin of replication and a TATA-box, P (68 bp), Q (39 bp), R (63 bp), and S (63 bp), containing TATA-like elements and the regulatory regions for early and late genes expression, is considered the infectious strain, shed in the urine of immunocompetent individuals [69,70,71]. Approximately 30 transcription factor binding sites are in silico predicted: SP1 has been the most extensively studied [72,73,74], although the additional role played by other transcription factors such as NF1, ETS1, NFκB, the glucocorticoid and progesterone receptors, and CREB were evidenced in several studies [73,75,76,77].

Similarly to JCPyV, the plausible instability of the archetype BKPyV NCRR could contribute to the development of the prototype strains, which is able to cause polyomavirus-associated nephropathy in kidney transplant recipients and hemorrhagic cystitis in hematopoietic stem cell transplant recipients [78,79,80,81]. The Dunlop strain, the most salient prototype strain, was isolated from a kidney transplant recipient with ureteral stenosis [82]. This strain displays three 68-bp tandem repeat within the NCRR (O-P-P-P-S arrangement) with respect to the archetype strain, carrying a single 68-bp motif. This strain showed less enhancer activity than the prototype strain, thus confirming the significance of the triplicated motifs on transcriptional regulation, and on viral infectious activity [83]. In fact, BKPyV strains isolated from kidney transplant recipients with rearranged NCRR showed higher viral gene expression and viral loads with more extensive pathogenicity [84].

Additional NCCR structures have been described for both viruses [85,86,87,88]. In particular, the presence of a common pattern of JCPyV NCCR rearrangement, such as the D-box deletion, can be considered a hallmark needed for the initial NCCR rearrangements critical co-factor for the development of PML in immunosuppressed individuals [88,89]. Besides the triplication of the P region, rearrangements of BKPyV NCCR involve the adjacent O and Q blocks. Differently, the S block is always retained, hence highlighting the importance of these nucleotide sequences [70]. NCCR mutations were also observed during in vitro JCPyV and BKPyV cultivation, confirming that NCRR variants could arise after prolonged propagation of the viruses in cells [71,85,90,91]. The mechanisms by which both viruses determine relevant human diseases are not established, but it is accepted that the regulation of gene expression in HPyVs plays a role in determining the viral tropism, and in the promotion of pathogenesis progression [92].

Little is known about the genetic diversity of the NCCRs from the novel HPyVs and the biological relevance in terms of viral transcription, replication, and possible pathogenic properties. In this review, we provide an overview of the mutations in the NCCR, which is defined as the sequence between the start codon of the *LT/sT* gene and the start codon of the *VP2* gene, of the novel HPyVs and their known effect on promoter activity. We discuss how NCCR rearrangements may affect the binding of putative transcription factors, and whether specific NCCR configurations are associated with disease.

## 3. KI and WU NCCR Variants

KIPyV has been mostly isolated from oral and respiratory specimens from (pediatric) patients with respiratory diseases that suffer from other viral and bacterial infections (reviewed in [93]). Whether KIPyV is a genuine respiratory pathogen or an opportunistic co-infector has not been established [93,94]. Seventy-two full-length NCCR sequences have been deposited in GenBank so far (Table S1). They contain the LT binding motifs, an AT-rich stretch and repeated sequences (Figure 1B and Table 2).

**Figure 2:**
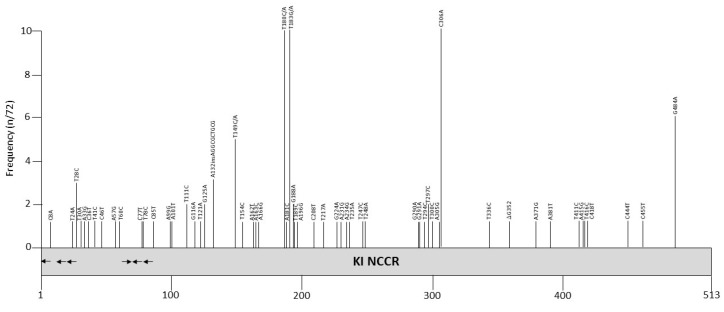
Mutations and their prevalence in variants of Karolinska Institute polyomavirus (KIPyV) noncoding control region (NCCR). The numbering of the NCCR is from early to late, with nucleotide 1 being the most proximal to the ATG start codon of the early genes and the most distal nucleotide, just upstream of the start codon of the *VP2* gene. The number of times a peculiar mutation is found in the different variants is given as frequency (with *n* the number of times the mutation was described/total NCCR variant sequences available). Putative Large T antigen (LT) binding motifs 5′-GRGGC-3′ (→) or 5′-GCCYC-3′ (←) are shown. The table summarizes the mutations, their location in the NCCR and their frequency. For details, see Table S1. Putative transcription factor binding sites are shown in Supplementary Table S2.

Most KIPyV NCCR sequences are obtained from nasopharyngeal swabs or aspirates, but also from blood form healthy blood donors [96] and from feces from a child with acute gastroenteritis [97]. The NCCR sequence of the Stockholm 60 isolate (Genbank accession number NC_009238; [7]; Figure S1) may represent the archetype NCCR because it is the most common sequence reported, and has been isolated from different biological samples in different parts of the world. Stockholm 60 KIPyV was originally isolated from respiratory tract specimens from a child. We found that 21 out of 48 isolates from nasopharyngeal aspirates of patients with respiratory symptoms or infections and 23 out of 38 isolates from healthy blood donors have the Stockholm 60 NCCR [96]. As described by us and others, NCCRs of other isolates, contain only minor point mutations scattered throughout the entire NCCR (Figure 2 and Table 3). Exceptions are the isolates Brisbane 001, Brisbane 005 and CU-255, whose NCCRs have the 10 bp AGGCGCTGCG insertion, and are clinical isolates obtained from respiratory tract (Table S1).

The KIPyV NCCR contains putative binding sites for several transcription factors (Table 4 and Table S2). The effect of this 10 bp AGGCGCTGCG insertion on KIPyV promoter activity or replication is not known, but the sequence contains a putative binding site for transcription factor AP4 [98]. AP4 is ubiquitously expressed, and can both activate and repress transcription [99,100]. Its effect on KIPyV NCCR has not been investigated. The point mutations remove or create putative binding sites for several transcription factors, including nuclear receptors, STAT proteins, HOXD, and POU the general transcription factors TBP and TFIID (see Table 3 in [101] for a detailed overview). We examined the effect of NCCR polymorphism in isolates from blood and nasopharyngeal samples on early and late promoter activity in HEK 293 cells [96]. These cells had previously been shown to give highest promoter activity of 10 different cell lines tested [102]. Eighteen isolates with a single nucleotide substitution were tested and revealed significant differences in early and late promoter activities for some of the isolates. One variant (NPA7d) had a mutation that destroyed a putative c-Myb binding motif compared to Stockholm 60 NCCR. Ectopic expression of c-Myb stimulated the early and late promoter activities of both Stockholm 60 and NPA7d, but there was no significant difference in c-Myb induced activation of the promoters [96]. Some of the mutations are located in putative LT binding sites and may therefore have an effect on promoter activity or/and viral DNA replication. It remains to be determined whether the NCCR may have an effect on the pathogenic properties of KIPyV because Stockholm 60 and Stockholm 60-like NCCRs have also been isolated from blood and respiratory specimens from healthy individuals, with no direct association between KIPyV and diseases having been established. Larger KIPyV NCCR rearrangements as seen for BKPyV and JCPyV NCCRs seem to be rare.

**Figure 3:**
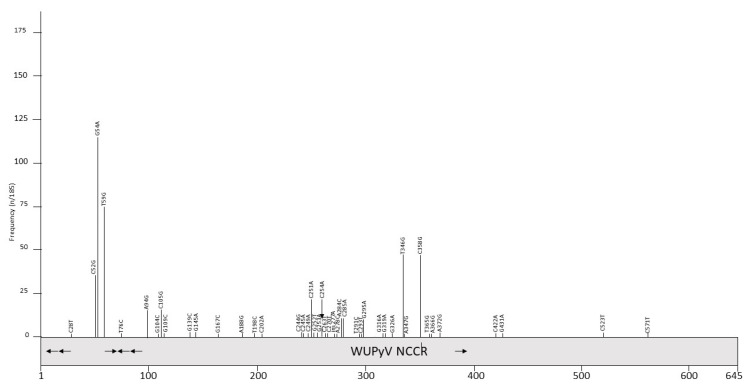
Mutations and their prevalence in variants of Washington University polyomavirus (WUPyV) noncoding control region (NCCR). The numbering of the NCCR is from early to late, with nucleotide being 1 the most proximal to the ATG start codon of the early genes and the most distal nucleotide, just upstream of the start codon of the *VP2* gene. The number of times a peculiar mutation is found in the different variants is given as frequency (with *n* the number of times the mutation was described/total NCCR variant sequences available). Putative Large T antigen (LT) binding motifs 5′-GRGGC-3′ (→) or 5′-GCCYC-3′ (←) are shown. The table summarizes the mutations, their location in the NCCR, and their frequency. For details see Table S1. Putative transcription factor binding sites are shown in Table S2.

A total of 185 partial or complete WUPyV NCCR sequences are available in the GenBank (Table S1). All strains have a NCCR of 645 bp, except variant J1, which has an insertion of one A at position 277 (Table S1), and contains an AT-rich stretch, GRGGC pentamers and repeated sequences of, respectively, 10 and 16 bp (Figure 1B and Table 2). Polymorphisms are predominantly in the NCCR part proximal to the early region (Figure 3 and Table 5). The most common point mutations are G54A and T59G. Both mutations are often present simultaneously. The substitution C52G is also common, but is always found in combination with the G54A mutation. The variants GD-WU709 and WU/Wuerzburg01/07 have C52T rather than C52G, whereas 12 variants have the triple mutation C52G/G54A/T59G. These three nucleotides are part of a sequence that is flanked, respectively, by 4 and 5 T residues; the triple substitution removes the putative binding site for transcription factor c-MYB, and creates motifs for TATA/TBP and retinoic acid receptor-related orphan receptor α [98]. The mutations A94G and C105G are also always simultaneously present, with the double mutations generating a remote sequence similarity with the binding motif of transcription factor AP1, though this does not seem to affect the binding of other putative factors (Table S2; [98]). Other common mutations include A284C and C285A, which are also found together except for the WU/Wuerzberg03/07 variant, which lacks the A284C substitution. A284/C285 are part of a putative site for RUNX1 (AML1; [98]), a transcription factor involved in hematopoiesis [103]. While G295A is found in 9 NCCR sequences, one strain (CQ6029/China_CQ/2014) had a G295C replacement. The CU_CHONBURI3 isolate from a patient with respiratory diseases had several unique point mutations. Overall, no typical mutations in specific specimens were detected, nor was an apparent correlation with a genotype and geographic regions. To the best of our knowledge, the effect of mutations on the WUPyV promoter activity has not been studied, nor have the consequences of viral replication been addressed. Whether mutations had an effect on putative transcription factor binding sites is also unknown, but because most mutations are single or few point mutations, they may not destroy or create novel binding sites.

## 4. MCPyV NCCR Variants

**Figure 4:**
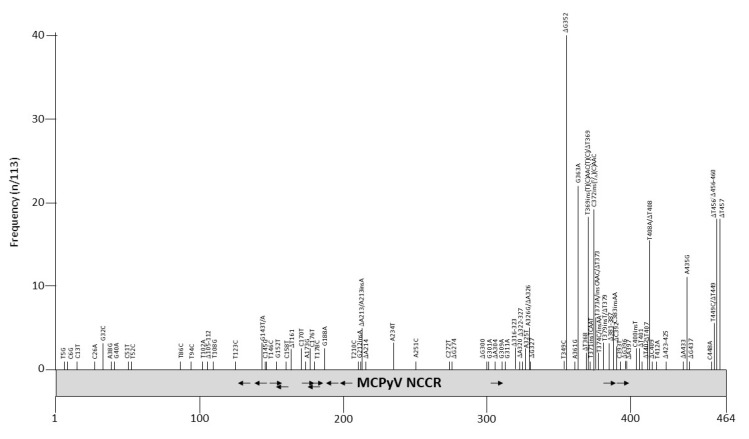
Mutations and their prevalence in variants of Merkel cell polyomavirus (MCPyV) noncoding control region (NCCR). The numbering of the NCCR is from early to late with nucleotide 1 being the most proximal to the ATG start codon of the early genes and the most distal nucleotide, just upstream of the start codon of the *VP2* gene. The number of times a peculiar mutation is found in the different variants is given as frequency (with *n* the number of times the mutation was described/total NCCR variant sequences available). Putative Large T antigen (LT) binding motifs 5′-GRGGC-3′ (→) or 5′-GCCYC-3′ (←) are shown. The table summarizes the mutations, their location in the NCCR, and their frequency. For details see Table S1. Putative transcription factor binding sites are shown in Supplementary Table S2.

NCCR rearrangements are described as a pivotal event in the onset of HPyVs-related pathology, as demonstrated for JCPyV and BKPyV, in which NCCRs not only control gene expression, but also serve as the main determinants in viral replication, containing the origin of DNA replication and transcription factor binding sites [104,105]. MCPyV is a major causative agent of the skin cancer Merkel cell carcinoma [9], but whether the NCCR can influence the outcome of the infection remains elusive. More than 100 partial or complete NCCR sequences are available from MCC and non-MCC tissue (Table S1). Mutations have been described throughout the entire NCCR, but especially in the late promoter part region (Figure 4 and Table 6). Nucleotides 360–425 of the MCPyV NCCR contain putative binding sites for transcription factors AP1, AP2, C/EBPα and β, EVI1, NFκB, c-Myb, p53, SOX5, TST-1, and SP1 (Table 4; [98]), although their binding has not been proven so far. Some of the mutations affect putative LT binding motifs, and may therefore interfere with transcription and replication of the viral DNA. Indeed, studies by the group of Chang and Moore showed that mutations in nucleotides G143, C145A, A173 and C176 abolished the replication of MCC isolates MCV339 and MCV350 in the presence of full-length LT [106,107]. The NCCR from MCC isolates FraMerk22 and FraMerk24 both contain the mutations G143T and C176T, whereas MCC isolate MKT-23 has the mutations G143A, C145G, and A173A, with MKT-32 carrying the transversion C146G. Since all these isolates are derived from MCC, they are replication deficient due to the expression of truncated LT and integration. None of the mutations identified by the work of the Chang-Moore group that abrogate MCPyV replication have been reported in non-MCC PyV isolates (see Table S1).

Whether a MCPyV variant with a particular NCCR architecture is associated with specific patient groups is not known. MCPyV with different NCCRs have been characterized in Merkel cell carcinoma samples (see Table S1), while for other diseases only a single sample of a particular individual was examined. Few studies have examined a larger cohort and/or different clinical samples from the same patient. Hashida, et. al. evaluated the genetic variability of MCPyV NCCR in skin swab specimens of healthy individuals with distinct ethnicities and geographic origins, identifying two major subtypes of MCPyV NCCR, subtypes I and II, with the presence or absence of a 25 bp tandem repeat (TGTCCTCCTCCCTTTGTAAGAGAAA) in the late promoter region. Based on the occurrences of two deletions (T368 and T369), and the 5 bp TCAAC insertion (compared to the consensus strain R17b), MCPyV strains were further assigned to five genotypes [108]. Delbue et.al. performed MCPyV NCCR molecular characterization on cerebrospinal fluid samples collected from patients affected by neurological disorders. The results obtained showed the presence of the MCPyV NCCR IIc strain, according to Hashida’s NCCR classification [109]. Prezioso, et al., studying the MCPyV NCCR from urine, plasma and rectal swabs recovered from immunosuppressed population, observed, in plasma and rectal swabs, as well as the occurrence of the MCPyV NCCR IIa-2 strain, which contains the 5 bp insertion and represents the predominant strain among white persons of European descent [110]. The deletion of nucleotide G352 is unique for the MCPyV isolates in plasma, urine and rectal swab specimens from HIV-1 patients, and has not been described in MCPyV isolates from other patient groups. In addition to the NCCR genotypes circulating within a HIV-1-positive population in the same study, Prezioso et al. evaluated the MCPyV NCCR alterations focusing on putative binding sites of cellular transcription factors, in order to verify whether mutations and/or rearrangements could fall in some binding sites [110]. The analysis of distal NCCR sequences (nucleotides 302–464) and the analysis of the relative putative binding site, revealed a high degree of homology with R17b strain in urine samples, whereas transitions, transversions, and single or double deletions were observed in plasma and rectal swabs (Table S1). Differently from JCPyV and BKPyV, in which the early proximal side of NCCR is highly conserved and the late proximal side undergoes rearrangements [111], insertions and deletions occurred in both the early and late proximal side of the MCPyV NCCR. More specifically, representative TCAAT and AAC insertions (nucleotide positions 5210–5211) were observed in both plasma and rectal swabs. Analysis of the putative binding site showed that the MCC350 NCCR sequence contains putative NF1, NFκB, TST-1, OCT1, AP-1, and TATA sites, already described within the NCCRs of other HPyVs [98,112]. In several strains obtained from MCPyV-positive plasma and rectal swabs samples, deletions, insertions, or single base substitutions fell within these putative binding sites, thus making predictable that some of these changes would not allow the identification of putative binding motifs, such as SP1 and/or p53, already described in the NCCR of other HPyVs [112]. Further studies are warranted in order to define the importance of these NCCR binding sites and to understand how their changes (mutations, insertions, or deletions) may influence in vivo MCPyV pathogenicity. In contrast to NCCR analysis conducted on rectal swabs from an HIV-1-positive population, which were characterized by the onset of transitions, transversions, and single or double deletions [110], MCPyV NCCR in stool samples from patients with hematological disorders exhibited a high degree of sequence stability, thereby suggesting that sequence rearrangements occurred rarely in the gastrointestinal anatomical site [113]. To date, although it is well documented that MCPyV DNA has been detected in the upper and lower respiratory tract specimens of children and adults and in immunocompetent and immunocompromised patients [114,115,116,117,118] and that the detection of MCPyV DNA was also observed in cystic fibrosis patient respiratory secretions [119,120], the respiratory NCCR structure organization has not yet been investigated.

The relative early and late promoter strength of seven MCPyV NCCR variants was compared in human dermal fibroblasts, and in the non-classical MCPyV-positive MCC cell line MCC13 [121]. All variants that had mutations compared to the consensus strain R17b (GenBank accession number HM011556) had a 10–50% lower basal early and late activity in both cell lines. However, the I strain described by Hashida et al. ([108]) had an approximately 30% higher early and late promoter activity and the early promoter of isolate MKL1, a MCC isolate [122], was approximately 40% stronger in the fibroblasts. The promoter activity of other variants has not been compared, nor has the effect of mutations on the viral life cycle and transforming potential of this oncovirus been examined.

## 5. HPyV 6 and HPyV7 NCCR Variants

Although HPyV6 and HPyV7 DNA is commonly present in the normal skin of healthy persons [10,123], HPyV6 and HPyV7 are associated with rash and pruritic skin eruption [124,125,126,127,128], HPyV7 DNA was found in 19/35 cholangiocarcinomas [129], while HPyV6 DNA has been detected in a few cases of keratoacanthomas, basal cell carcinomas, squamous cell carcinomas and trichoblastomas [130,131]. HPyV6 DNA was detected in 1/234 cerebrospinal fluid samples and 1/1016 serum samples of healthy blood donors [109,132]. HPyV6 DNA prevalence was much higher in tonsil brushing samples from immunocompetent children and adults than HPyV7 DNA (113/689 versus 6/689). HPyV6 and HPyV7 DNA prevalence and copy number were significantly higher in skin swabs collected from lesional and non-lesional skins of 86 Japanese patients with inflammatory skin diseases and mycosis fungoides compared with specimens from 149 healthy control individuals [133]. HPyV6 and HPyV7 were detected in 1/55 skin specimens from cutaneous T-cell lymphoma patients [29]. Despite the presence of HPyV6 and HPyV7 DNA in samples of various disorders, it remains to be established whether these viruses play a direct role in causing such skin conditions.

**Figure 5:**
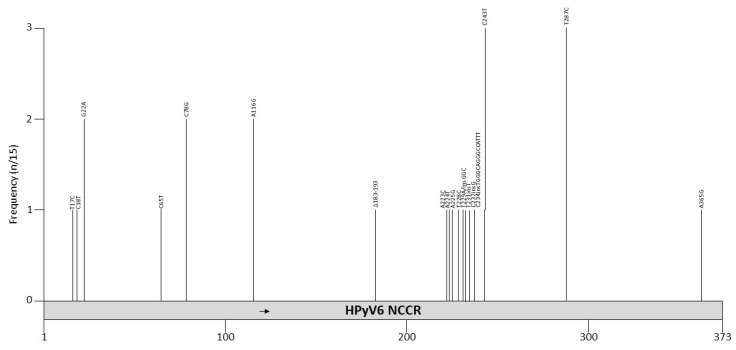
Mutations and their prevalence in variants of HPyV6 noncoding control region (NCCR). The numbering of the NCCR is from early to late with nucleotide 1 being the most proximal to the ATG start codon of the early genes and the most distal nucleotide, just upstream of the start codon of the *VP2* gene. The number of times a peculiar mutation is found in the different variants is given as frequency (with *n* the number of times the mutation was described/total NCCR variant sequences available). Putative Large T antigen (LT) binding motifs 5′-GRGGC-3′ (→) or 5′-GCCYC-3′ (←) are shown. The table summarizes the mutations, their location in the NCCR, and their frequency. For details see Table S1. Putative transcription factor binding sites are shown in Supplementary Table S2.

Seventeen HPyV6 NCCR sequences are deposited in GenBank. Four of them are sequences obtained from HPyV6 DNA amplified in sewage (H6-cg-A2.f, B159.4, U43.1 and U43.3), six are from healthy skin (606b, 607a, 607b, 609a, 614a, and 627a), two are from bile samples (Bile-72 and Bile-81), and two are combined nose and throat samples from kidney transplant patients (QLD-49Br and QLD-61Br). One sample was obtained from pruritic skin lesion (UTSW6.1), one from a lymph node from a patient with an angiolymphoid hyperplasia with Kimura disease (LN1), and one from a nasopharyngeal aspirate of a child with respiratory tract infections (BJ376) (see Table S1 for details and references). Identical HPyV6 NCCRs were found in healthy skin, along with bile from patients with malignant biliary obstruction, combined nose and throat specimens from kidney transplant patients and a nasopharyngeal sample of a child with respiratory infection (Table S1). Two clinical samples (UTSW6.1 from pruritic skin and LN1 from the lymph node of a patient with Kimura disease) and the DNA amplified from sewage water had mutations compared to the reference strain. The mutation spectrum is shown in Figure 5 and Table 7. The UTSW6 isolate had a deletion of nucleotides 183–193 (CAAAGGTCAAA), a mutation of nucleotides 223–229 (except 228), and insertions of GGC and of TGGGCAGGGCCATTT distal of these point mutations. The 11 bp deletion removes binding motifs for AP1 and CREB, while the 15 bp insertion adds a putative SP1 and p53 binding site. Other putative binding sites are shown in Table 4 and Table S2. The point mutations affect an AT-rich region but no putative binding motifs are predicted in this sequence [98], which may affect viral replication, as this region is part of the predicted ori [134]. Based on the limited available HPyV6 NCCR sequences, no specific HPyV6 NCCR is associated with disease. The effect of mutations in the NCCR on the promoter activity and viral life cycle has not been tested.

**Figure 6:**
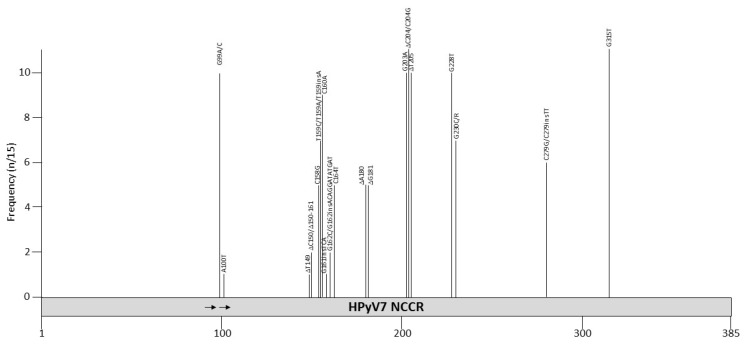
Mutations and their prevalence in variants of HPyV7 noncoding control region (NCCR). The numbering of the NCCR is from early to late with nucleotide 1 being the most proximal to the ATG start codon of the early genes and the most distal nucleotide, just upstream of the start codon of the *VP2* gene. The number of times a peculiar mutation is found in the different variants is given as frequency (with *n* the number of times the mutation was described/total NCCR variant sequences available). Putative Large T antigen (LT) binding motifs 5′-GRGGC-3′ (→) or 5′-GCCYC-3′ (←) are shown. The table summarizes the mutations, their location in the NCCR, and their frequency. For details see Table S1. Putative transcription factor binding sites are shown in Supplementary Table S2.

Sequences of 15 different HPyV7 NCCR isolates are available in GenBank (variants 707a, 707b, 713a, 713b, 715b, 727a, CRC01, PITT1, PITT2, UTSW7.1, PLA1, PLA2, MUQ, URI, and BIO). Six are from healthy skin specimens, while nine are clinical samples from patients, including skin lesions from lung or renal transplant patients with rash and pruritus, and skin lesions from patients with pruritic and dyskeratotic dermatoses (Table S1 and references therein). The length of these NCCRs varies from 371 bp (PITT2 isolate) to 399 bp (PITT1 isolate). DNA of these two variants was isolated from the skin of lung transplant patients with a rash [124]. Five isolates from the same patient had an NCCR of 381 bp (BIO, MUQ, PLA1, PLA2, URI), five had a 383 bp NCCR (707a, 707b, 715b, 727a, UTSW7.1), two had a 385 bp NCCR (713a and 713b), and one had a NCCR of 387 bp (CRC01). No repeated sequences are present (Table 2). The mutations in the different HPyV7 variants are concentrated in the central part of the NCCR (Figure 6 and Table 8). The consensus is the nucleotide sequence that was present in the majority of the 15 available sequences, with the nucleotide numbering based on the HPyV7 reference strain R713a (GenBank accession number NC_014407=713). Most mutations are point mutations, whereas PITT1 also contains the insertion ACAGGATATGAT and PITT2 has a deletion removing nucleotides 150–161 (CTGGGTTACTGG). The insertion contains putative binding sites for the transcription factors ETS1, GATA1/2/3 and EVI1, whereas the deletion removes possible GATA2 and CDP binding motifs [98]. EVI1, CDP and GATA3 are expressed in the skin, while ETS1, GATA1, and GATA2 are not or weakly expressed in skin [100]. Putative binding sites for transcription factors in the HPyV7 NCCR are summarized in Table 4 and Table S2.

The early promoter activity of the PITT1 and PITT2 variants was significantly higher than the activity of the reference strain in the colon adenocarcinoma cell line SW480, whereas a tendency to lower activity in human embryonal kidney HEK293 cells was observed [112]. The promoter activity was not examined in skin cells, although these variant were originally isolated from the skin [124]. Colon and kidney cells may not be authentic host cells because no HPyV7 LT expression was detected in 10 normal and 94 malignant colon samples, and in 10 normal and 65 renal cancers [135] and so far there are no reports of HPyV7 DNA in these organs. A transversion of A to T in the putative 5′-GAGGC-3′ LT motif was reported (Figure 6), although the effect on viral replication has not been exploited.

Interestingly, the NCCR of the recently isolated QPyV DNA shows >80% identity with the HPyV7 NCCR (Figure S3), while the complete genome is 81% identical with HPyV7 [18].

## 6. TSPyV NCCR Variants

**Figure 7:**
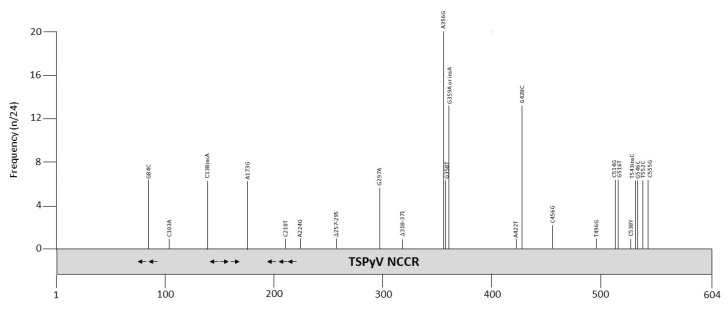
Mutations and their prevalence in variants of Trichodisplasia spinulosa polyomavirus (TSPyV) noncoding control region (NCCR). The numbering of the NCCR is from early to late with nucleotide 1 being the most proximal to the ATG start codon of the early genes and the most distal nucleotide, just upstream of the start codon of the *VP2* gene. The number of times a peculiar mutation is found in the different variants is given as frequency (with *n* the number of times the mutation was described/total NCCR variant sequences available). Putative Large T antigen (LT) binding motifs 5′-GRGGC-3′ (→) or 5′-GCCYC-3′ (←) are shown. The table summarizes the mutations, their location in the NCCR, and their frequency. For details see Table S1. Putative transcription factor binding sites are shown in Supplementary Table S2.

Twenty-four TSPyV NCCR sequences are deposited in the GenBank (Table S1). Most samples are derived from skin spicules, but also a nasopharyngeal aspirate from a heart transplant patient, a heart from a myocarditis patient, and the CSF and serum of immunosuppressed patients contained TSPyV DNA. The NCCRs of the non-spicule isolates were identical or quasi identical with isolates from skin spicules. Most mutations are point mutations (Figure 7 and Table 9), but two skin spicule isolates (0602 and 1312) had deletions of 54 and 38 bp, respectively [136]. The relative promoter activity of these NCCR variants has not been examined, nor has the effect of mutations on the viral life cycle been investigated. The 39 bp deletion removes putative binding sites for AP1, SOX5, HNF3, OCT1, TATA/TBP, STAT, glucocorticoid receptor, retinoic acid receptor-related orphan receptor α, and CREB, whereas 54 bp deletion possesses possible binding sites for ARNT, AP1, AP2, AP4, CREB/ATF, CAAT, E2F, ELK, EVI1, GATA1/2/3, NHLH1, MYB, MYC, MYOD, NFκB, OCT1, PAX5, TST1, and USF [98]. While most of these factors are expressed in the skin, MYOD, PAX5, GATA1, and GATA2 seem to be absent in the skin [100]. However, the binding of these transcription factors and their possible role in regulating TSPyV transcription remain to be proven. The TSPyV NCCR contains several putative LT binding motifs, and mutations in some of them have been reported (Figure 7). Whether they have an effect on viral replication has not been tested.

## 7. HPyV 9 NCCR Variants

HPyV9 was originally detected in the serum and urine from a renal transplant patient under immunosuppressive treatment [12]. Shortly after, HPyV9 DNA was isolated from the facial surface of a Merkel cell carcinoma patient and tentatively named Institute Pasteur polyomavirus (IPPyV) [137]. The genome of IPPyV only differs by two nucleotides from HPyV9, hence IPPyV is a variant of HPyV9. Yet, none of these mutations are within the NCCR (Table S1; [137]). The HPyV9 isolate M149 from tonsils has an identical NCCR sequence as the original HPyV9 isolate (GenBank accession MH844627). An HPyV9 isolate (UF-1 isolate) from the blood of an AIDS patients displays an eight base-pair deletion, a 13 base-pair insertion and 24 point mutations in its NCCR [138]. These NCCR rearrangements created putative SP1 binding sites in the late promoter. We compared the basal early and late promoter activity of the original HPyV9 strain and the UF-1 clinical isolate in the human cell lines BEL7402, C33A, HEK293, HeLa, SK-N-BE, SW480, and U2OS. We found that the UF-1 early promoter was stronger in all cell lines except in U2OS, and the UF-1 late promoter was stronger in all cell lines except in C33A and HeLa cells [139]. The effect of LT on early and late promoter activity was monitored in BEL7402, HEK293 and HeLa cells. Whereas the UF-1 late promoter activity was more potently stimulated than the HPyV9 late promoter by LT in all three cell lines tested, a stronger LT-induced activation of the UF-1 early promoter compared to the HPyV9 early promoter was only observed in HEK293 cells. The mutations in the UF-1 NCCR generate two putative SP1 binding sites in the distal part of the late promoter. Mutating these two SP1 sites did not have an effect on the basal early promoter activity, but increased basal late promoter 2-fold. Disruption of these SP1 sites had also no effect on LT-induced early promoter activity, but reduced late promoter activity 7-fold compared to non-mutated late UF-1 promoter activity. Our results showed that the promoter activity of the clinical isolate UF-1 is stronger and more potently induced by LT compared with the promoter of the original HPyV9 isolate. A later study confirmed that the UF-1 promoter was stronger than the promoter of the original isolated HPyV9 in HEK293 and the lung carcinoma A549 cells [112]. Whether the rearrangements in the UF-1 NCCR may affect the life cycle and possible pathogenic properties of the virus remains to be determined. Additional putative transcription factor binding sites are summarized in Table 4 and Table S2.

## 8. HPyV10 NCCR Variants

**Figure 8:**
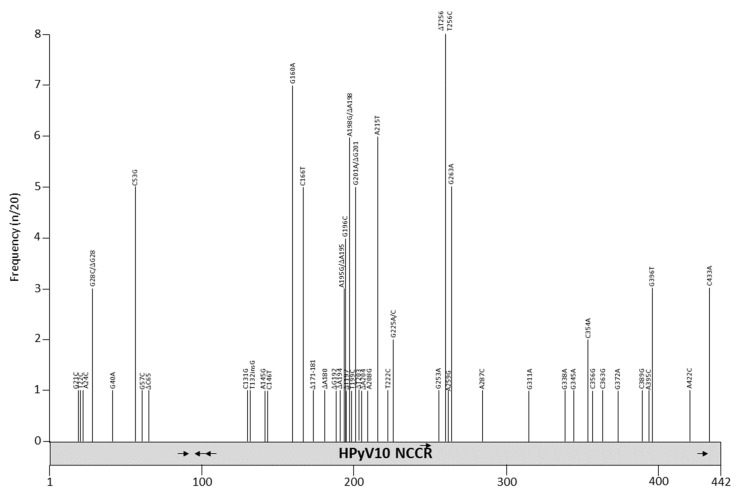
Mutations and their prevalence in variants of HPyV10 noncoding control region (NCCR). The numbering of the NCCR is from early to late with nucleotide 1 being the most proximal to the ATG start codon of the early genes and the most distal nucleotide, just upstream of the start codon of the *VP2* gene. The number of times a peculiar mutation is found in the different variants is given as frequency (with *n* the number of times the mutation was described/total NCCR variant sequences available). Putative Large T antigen (LT) binding motifs 5′-GRGGC-3′ (→) or 5′-GCCYC-3′ (←) are shown. The table summarizes the mutations, their location in the NCCR, and their frequency. For details see Table S1. Putative transcription factor binding sites are shown in Supplementary Table S2.

Twenty NCCR sequences are available in GenBank (Table S1), with the length ranging from to 430 to 442 bp. The original isolates MWPyV (NC_018102) and MA095 (JQ898291), both from feces [140], are identical, but contain an 11 bp deletion compared to the other variants (Table S1, Figure 8 and Table 10). The NCCRs of isolates ww10, TEDDY-01, QLDMW04 and QLDMW10 are identical, although they were derived from different specimens from different patients. The ww10 isolate was detected in a condyloma specimen from a patient with warts, hypogammaglobulinemia, infections, and myelokathexis (WHIM) syndrome [13], QLDMW04 and 010 are from respiratory samples [141], and TEDDY_01 is from feces (direct submission to GenBank; accession number KC549591). Point mutations are dispersed throughout the NCCR for the other isolates. The 11bp deletion (ATTGTTGGCAA) contains possible binding sites for CDP and SOX5 [98]. CDP is ubiquitously expressed, but SOX5 is enriched in testis [100]. Other possible transcription factors that may bind the HPyV10 NCCR are given in Table 4 and Table S2. It is not known whether HPyV10 is associated with a disease, and the biological consequence of NCCR mutations remains elusive.

## 9. STLPyV NCCR Variants

**Figure 9:**
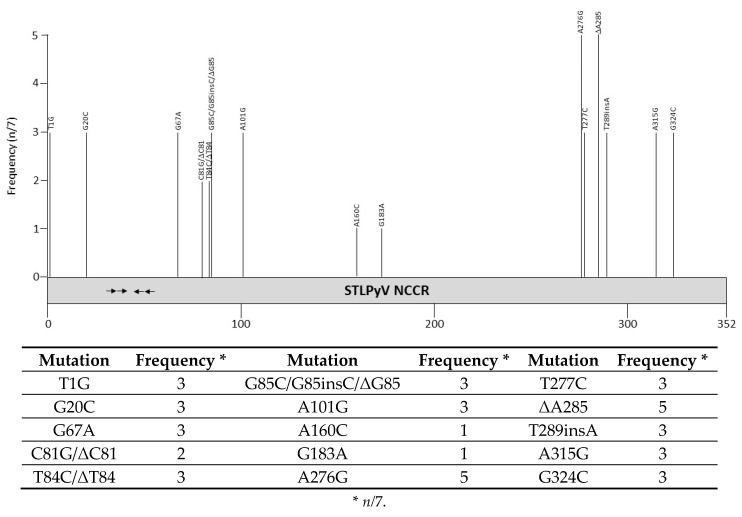
Mutations and their prevalence in variants of Saint Louis polyomavirus (STLPyV) noncoding control region (NCCR). The numbering of the NCCR is from early to late with nucleotide 1 being the most proximal to the ATG start codon of the early genes and the most distal nucleotide, just upstream of the start codon of the *VP2* gene. The number of times a peculiar mutation is found in the different variants is given as frequency (with *n* the number of times the mutation was described/total NCCR variant sequences available). Putative Large T antigen (LT) binding motifs 5′-GRGGC-3′ (→) or 5′-GCCYC-3′ (←) are shown. The table summarizes the mutations, their location in the NCCR, and their frequency. For details see Table S1. Putative transcription factor binding sites are shown in Supplementary Table S2.

Sequences of 7 STLPyV NCCRs are available in GenBank (Table S1). These variants were discovered in feces, respiratory swab or peri-anal warts [14,26,27,142,143]. Point mutations and one bp deletions have been observed (Figure 9 and Table S1), although the biological implications on promoter activity and viral replication have not been examined, nor has their possible role in disease been defined. So far, no variants with mutations in the possible LT binding motifs have been isolated. Putative binding sites for transcription factors in the STLPyV, NCCR are shown in Table 4 and Table S2.

## 10. HPyV12, NJPyV, LIPyV and QPyV Variants

The NCCR sequence of two HPyV12 isolates is known. One of them carries a 26 bp deletion in the distal part of the late promoter (nucleotides 297–322) (Table S1; [102]). This deletion reduces the early and late promoter activity in 10 different human cell lines tested, except for the early promoter activity in BEL7402 and HEK293 cells, in which a significantly higher activity was measured compared with the early promoter activity of the original HPyV12 isolate [102]. The deletion eliminates putative c-MYB, CREB and AP4 binding sites, but it is not known whether these transcription factors actually bind the HPyV12 promoter.

Only one human and one feline LIPyV isolate have been reported, and they differ by four-point mutations (Table S1). No NJPyV and QPyV NCCR variants have been described thus far. Putative binding sites for transcription factors in the HPyV12, NJPyV and LIPyV NCCR are summarized in Table 4 and Table S2.

## 11. Conclusions

Similar to BKPyV and JCPyV, novel HPyV isolates with mutations in their NCCR are commonly detected in human samples. However, for the most recently described PyVs isolated from human specimens, none or very few isolates have been reported, and large deletions and/or duplication are lacking. Our knowledge of the effect of mutations in the NCCR on viral promoter activity and viral replication is incomplete because only a few studies have addressed the effect of NCCR mutations on the promoter activity, while the impact on viral replication has not been examined. Replication studies have been hampered by the lack of permissive cell systems for all novel HPyVs, except dermal fibroblasts which support productive MCPyV infection [144]. The HPyV NCCRs contain a plethora of binding motifs for host cell proteins, but their binding to the NCCR has not been confirmed. Chromatin immunoprecipitation assays at the early and late stages of infection may allow for the identification of transcription factors involved in early and late expression. Another unsolved question for most of the novel HPyVs is whether they are associated with specific diseases. Apart from MCPyV and its etiologic role in MCC, TSPyV as the causative agent of trichodysplasia spinulosa [9,145,146,147], and HPyV6 and HPyV7 with pruritic skin eruption in immunocompromised patients [124,125,126,127,128], firm evidence for pathogenic properties of the other novel HPyVs is lacking. So, far no specific MCPyV, HPyV6, HPyV7, and TSPyV NCCR variants seem to be associated with disease because virus variants with (quasi) identical NCCRs were also detected in samples from healthy individuals. Studies on different patient groups are required to unveil possible novel HPyV-associated diseases, as more NCCR sequences from larger and different patient cohorts are required to establish a possible connection between the genetic diversity of the NCCR and disease. The biological consequences of NCCR mutations for the viral life cycle warrants further investigation.

## Figures and Tables

**Table 1:** The novel human polyomaviruses, their original source of isolation and their association with human diseases.

Virus	Original Source	Associated Disease	Reference
KIPyV	Nasopharyngeal aspirate	None	[7]
WUPyV	Bronchoavelar lavage	None	[8]
MCPyV	Merkel cell carcinoma	None	[9]
HPyV6	Healthy skin	Pruritic skin eruption in immunocompromised patients	[10]
HPyV7	Healthy skin	Pruritic skin eruption in immunocompromised patients	[10]
TSPyV	Trichodysplasia spinulosa spicules	Trichodysplasia spinulosa	[11]
HPyV9	Serum from renal transplant recipient	None	[12]
HPyV10	Condyloma specimens from a patient with WHIM * syndrome	None	[13]
STLPyV	Stool sample from a healthy 15-month-old child	None	[14]
HPyV12	Liver sample from patient with malignant disease	None	[15]
NJPyV	Muscle biopsy from a pancreatic transplant patient	None	[16]
LIPyV	Skin swab	None	[17]
QPyV	Stool sample from 85-year old hospital patient	None	[18]

* warts, hypogammaglobulinemia, infections, and myelokathexis.

**Table 2:** Repeat sequences in the noncoding regions of the novel human polyomaviruses.

HPyV	Sequence	Position	Length	Remarks
KIPyV	CGTGAAGCCAACTTCCTG-GGC CGTG-AGCCAGCTTCCTGCGGC	251–271; 272–292	21	Imperfect DR *
WUPyV	GCCCTTTGTA ATGTTGTGACATCTCC	319–328; 389–398 479–494, 548–563	10 16	-
MCPyV	CAGAGGCCTC AACTTTTTTTC	147–156; 170–179 328–338; 370–380	10 11	Palindrome
HPyV6	No repeats	-	-	
HPyV7	No repeats	-	-	-
TSPyV	GAAATGAA	34–41; 42–49	7	DR
HPyV9	CTGTGGTAT	275–283; 284–292	9	DR
HPyV10	GCTATTGTTGGCAA	168–181;182–195	14	DR
STLPyV	No repeats	-	-	-
HPyV12	GTTCC CAGGCAGACGGCCAAGTTCC	203–207; 229–233 208–227; 228–248	5 20	GTTCC is part of larger repeat DR separated by 1 nucleotide
NJPyV	No repeats	-	-	-
LIPyV	No repeats	-	-	-
QPyV	No repeats	-	-	-

* DR = direct repeat. Repeat sequences were identified using the programs Tandem Repeat finder program [95] and the repeat sequence finding tool from Novoprolabs (novoprolabs.com/tools/repeat-sequence-finder).

**Table 3:** Frequency of mutations in the noncoding control region of Karolinska Institute polyomavirus.

Mutation	Frequency *	Mutation	Frequency	Mutation	Frequency
C8A	1	A132insAGGCGCTGCG	3	T248A	1
T24A	1	T149C/A	4	G290A	1
T28C	3	T154C	1	G291A	1
T30A	1	A162T	1	T294C	1
A33G	1	A163C	1	T297C	2
C36T	1	A166G	1	T300C	1
T41C	1	T180C/A	10	A305G	1
C46T	1	A181C	1	C306A	10
A57G	1	T183G/A	10	T336C	1
T60C	1	G188A	2	ΔG352	1
C77T	1	T189C	1	A371G	1
T78C	1	A196G	1	A381T	1
C85T	1	C208T	1	T411C	1
A99G	1	T217A	1	A415G	1
A101T	1	G224A	1	T416C	1
T111C	2	A231G	1	C418T	1
G116A	1	A234G	1	C444T	1
T121A	1	T235A	1	C455T	1
G125A	2	T247C	1	G484A	6

* *n*/72.

**Table 4:** Number of putative transcription factor binding sites in the noncoding control region of the novel human polyomaviruses. Prediction based on TFBIND tool [98].

Transcription Factor	KIPyV	WUPyV	MCPyV	HPyV6	HPyV7	TSPyV	HPyV9	HPyV10	STLPyV	HPyV12	NJPyV	LIPyV	QPyV
AML1	6	3	3	6	4	6	6	3	4	2	7	4	3
ARNT	2	0	0	3	2	4	2	2	2	5	4	2	2
AP1	8	9	10	6	8	14	8	7	10	4	5	5	5
AP2	12	13	6	7	6	6	5	6	5	5	7	3	0
AP4	8	12	5	0	6	6	5	5	9	4	9	3	3
ATF/CREB	7	11	6	4	8	14	5	6	10	2	5	3	8
CAAT	7	7	3	5	5	7	8	9	4	3	6	4	5
C/EBP	7	9	8	9	9	12	13	9	4	7	7	11	0
CDP	2	2	0	1	1	1	2	4	3	3	2	1	1
E2F	9	10	4	4	5	14	9	6	8	6	7	5	2
E47	7	8	1	2	5	3	5	4	4	2	6	2	2
ELK1	10	8	9	5	4	12	8	8	3	6	8	6	5
EVI1	3	6	4	3	4	9	7	3	4	4	5	5	5
GATA-1	7	12	9	8	6	16	15	9	6	6	7	10	8
GATA-2	6	8	5	3	5	13	8	6	4	8	3	5	7
GATA-3	5	0	2	1	5	8	6	1	0	0	1	4	3
GR	2	0	0	1	2	3	0	2	1	3	0	1	3
HNF3	3	4	2	2	1	4	1	3	4	1	2	0	2
IRF	5	3	6	4	4	2	3	4	0	2	4	3	3
MYB	6	5	6	7	0	4	6	6	3	7	2	7	1
MYC/MAX	5	7	1	2	2	6	3	2	2	3	4	1	1
MYOD	9	9	3	3	5	7	8	6	8	8	5	6	2
NF1	5	5	3	1	1	4	1	6	4	3	4	2	1
NFκB	2	3	5	2	1	5	7	5	0	1	1	1	1
NHLH1	2	2	2	1	1	1	0	0	1	0	1	1	0
OCT1	9	17	11	8	7	16	16	6	8	8	13	9	9
p53	7	8	5	4	3	8	9	5	1	11	10	5	3
PAX2	3	1	0	3	1	4	2	1	2	5	5	1	4
PAX5	7	4	5	2	3	3	8	1	5	2	3	1	3
PAX6	1	2	0	1	0	0	2	0	1	1	2	0	2
SOX5	3	7	4	2	4	8	2	5	7	2	5	4	4
RORα	7	4	3	4	6	5	4	1	2	6	2	0	3
SP1	12	12	14	6	8	13	10	9	7	11	8	11	11
SREBP	3	1	1	1	2	1	1	3	5	0	2	1	3
SRF	3	5	1	4	2	7	5	3	0	3	3	1	5
STAT	3	6	2	2	3	7	2	3	3	2	5	1	0
T3R	6	6	1	3	1	1	3	2	3	2	3	0	4
TBP	5	10	5	5	5	5	5	4	4	6	2	3	5
TST-1	3	5	4	2	0	4	2	2	1	0	1	2	1
USF	12	12	3	6	7	13	7	8	5	4	8	2	7
YY1	3	3	4	3	6	6	5	1	1	3	2	2	3

**Table 5:** Frequency of mutations in the noncoding control region of Washington University polyomavirus.

Mutation	Frequency *	Mutation	Frequency	Mutation	Frequency
C28T	1	C245A	1	G316A	1
C52G	33	C249A	1	G319A	1
G54A	114	C251A	18	G326A	2
T76C	1	G252T	1	T346G	17
A94G	15	G253A	1	A347G	1
G104C	1	C254A	5	C358G	20
C105G	15	C263T	1	T365G	1
G109C	1	C270T	1	A366G	1
G139C	2	ins277A	1	A372G	5
G145A	3	A278C	1	C422A	1
G167C	1	A284C	10	G431A	1
A188G	1	C285A	10	C523T	1
T198C	1	T291C	1	C571T	1
C202A	1	C293T	1		
C244G	2	G295A	8		

* *n*/185.

**Table 6:** Frequency of mutations in the noncoding control region of Merkel cell polyomavirus.

Mutation	Frequency *	Mutation	Frequency	Mutation	Frequency
T5G	1	G188A	2	T371insCAAT	1
C6G	1	T210C	1	C372ins(^T^/_A_)(C)AAC	19
C13T	1	G212insA	2	T373A/insCAAC/ΔT373	6
C26A	1	ΔA213/A213insA	5	T374C/insAA	2
G32C	3	ΔA214	1	T379insT/ΔT379	3
A38G	1	A234T	3	Δ381–387	3
G40A	1	A251C	1	C383insAA	5
C51T	1	C272T	1	ΔC392	3
T52C	1	ΔG274	1	C393T	1
T86C	1	ΔG300	1	ΔG396	1
T94C	1	G301A	1	ΔA397	1
T102A	1	ΔA304	1	C400insT	2
Δ105–112	2	G309A	1	ΔT401	2
T108G	1	G311A	1	ΔT402	1
T123C	1	Δ316–323	2	ΔT407	3
G143T/A	3	ΔA320	1	T408A/ΔT408	14
C145G	1	Δ322–327	1	ΔC409	1
T146C	1	A325T	2	T412A	1
G152T	2	A326G/ ΔA326	3	ΔT423–425	1
C158T	1	T349C	1	ΔA433	1
ΔT161	2	ΔG352	40	A435G	17
C170T	2	A361G	1	C448A	1
A173G	1	G363A	22	T449C/ΔT449	7
C176T	2	ΔT368	3	ΔT456/Δ456–460	18
T178C	1	T369ins(T)(C)AAC(T)(C)/ ΔT369	14	ΔT457	17

* *n*/113.

**Table 7:** Frequency of mutations in the noncoding control region of HPyV6.

Mutation	Frequency *	Mutation	Frequency	Mutation	Frequency
T17C	1	Δ183–193	1	T231insG	1
C18T	1	A223C	1	C232insG	1
G22A	2	A224T	1	C234insTGGGCAGGGCATTT	1
C65T	1	A225G	1	C243T	3
C78G	2	T228C	1	T287C	3
A116G	2	T229A/insGGC	1	A356G	1

* *n*/17.

**Table 8:** Frequency of mutations in the noncoding control region of HPyV7.

Mutation	Frequency *	Mutation	Frequency	Mutation	Frequency
G99A/C	10	G161insTCA	1	ΔT205	10
A100T	1	G162C/insACAGGTATGAT	2	G228T	10
ΔT149	1	C164T	5	G230C/R	7
ΔC150/Δ150–161	2	ΔA180	5	C279G/insTT	6
C158G	5	ΔG181	5	G315T	11
T159(C)(A)/insA	7	G203A	10		
G160A	9	C204G/ΔC204	11		

* *n*/18.

**Table 9:** Frequency of mutations in the noncoding control region of Trichodisplasia spinulosa polyomavirus.

Mutation	Frequency *	Mutation	Frequency *	Mutation	Frequency *
G84C	6	Δ318–371	1	C514G	6
C103A	1	A356G	20	G516T	6
C138insA	6	G358T	6	C538Y	1
A173G	6	G359A/G359insA	13	T543insC	6
C210T	1	A422T	1	G546C	6
A224G	1	G428C	13	T552C	6
Δ257–295	1	C456G	2	C555G	6
G297A	5	T496G			

* *n*/24.

**Table 10:** Frequency of mutations in the noncoding control region of HPyV10.

Mutation	Frequency *	Mutation	Frequency	Mutation	Frequency
G21C	1	ΔA180	1	G263A	5
T22C	1	ΔG192	1	A287C	1
A24C	1	ΔA194	1	G311A	1
G28C/ΔG28	3	A195G/ΔA195	4	G338A	1
G40A	1	G196C	4	G345A	1
C53G	5	A198G/ΔA198	6	C354A	2
G57C	1	G201A/ΔG201	5	C356G	1
ΔC65	1	ΔT203	1	C363G	1
C131G	1	ΔA204	1	G372A	1
T132insG	1	A208G	1	C389G	1
A145G	1	A215T	6	A395C	1
C146T	1	T222C	1	G396T	3
G160A	7	G225A/C	2	A422	1
C166T	5	T256C/ΔT256	8	C433A	3
Δ171–181	1	A259A	1		

* *n*/20.

## Supplementary Materials

The following are available online at https://www.mdpi.com/1999-4915/12/12/1406/s1, Table S1: mutations in the NCCRs of the novel HPyV. Table S2: Putative transcription factor binding sites in the NCCR of the novel HPyVs. Figure S1: NCCR sequence of the novel HPyVs. Figure S2: alignment of the NCCR from the novel HPyVs. Figure S3: Alignment of the HPyV7 and QPyV NCCRs.

## Abbreviations

bp	Base-pairs
HPyV	Human polyomavirus
IPPyV	Institute Pasteur polyomavirus
KIPy	Karolinska Institute polyomavirus
LIPyV	Lyon IARC polyomavirus
LT	Large T antigen
MCC	Merkel cell carcinoma
MCPyV	Merkel cell polyomavirus
MPyV	Mouse polyomavirus
NCCR	Non-coding control region
NJPyV	New Jersey polyomavirus
PML	Progressive multifocal leukoencephalopathy
QPyV	Quebec polyomavirus
sT	Small T antigen
STLPyV	Saint Louis polyomavirus
TSPyV	Trichodisplasia spinulosa polyomavirus
WUPyV	Washington University polyomavirus

## References

[1] DeCaprio J.A., Garcea R.L. (2013). A cornucopia of human polyomaviruses. Nat. Rev. Microbiol..

[2] Moens U., Krumbholz A., Ehlers B., Zell R., Johne R., Calvignac-Spencer S., Lauber C. (2017). Biology, evolution, and medical importance of polyomaviruses: An update. Infect. Genet. Evol..

[3] Peretti A., FitzGerald P.C., Bliskovsky V., Pastrana D.V., Buck C.B. (2015). Genome Sequence of a Fish-Associated Polyomavirus, Black Sea Bass (Centropristis striata) Polyomavirus 1. Genome Announc..

[4] Buck C.B., Van Doorslaer K., Peretti A., Geoghegan E.M., Tisza M.J., An P., Katz J.P., Pipas J.M., McBride A.A., Camus A.C. (2016). The Ancient Evolutionary History of Polyomaviruses. PLoS Pathog..

[5] Gardner S.D., Field A.M., Coleman D.V., Hulme B. (1971). New human papovavirus (B.K.) isolated from urine after renal transplantation. Lancet.

[6] Padgett B.L., Walker D.L., ZuRhein G.M., Eckroade R.J., Dessel B.H. (1971). Cultivation of papova-like virus from human brain with progressive multifocal leucoencephalopathy. Lancet.

[7] Allander T., Andreasson K., Gupta S., Bjerkner A., Bogdanovic G., Persson M.A., Dalianis T., Ramqvist T., Andersson B. (2007). Identification of a third human polyomavirus. J. Virol..

[8] Gaynor A.M., Nissen M.D., Whiley D.M., Mackay I.M., Lambert S.B., Wu G., Brennan D.C., Storch G.A., Sloots T.P., Wang D. (2007). Identification of a novel polyomavirus from patients with acute respiratory tract infections. PLoS Pathog..

[9] Feng H., Shuda M., Chang Y., Moore P.S. (2008). Clonal integration of a polyomavirus in human Merkel cell carcinoma. Science.

[10] Schowalter R.M., Pastrana D.V., Pumphrey K.A., Moyer A.L., Buck C.B. (2010). Merkel cell polyomavirus and two previously unknown polyomaviruses are chronically shed from human skin. Cell Host Microbe.

[11] van der Meijden E., Janssens R.W., Lauber C., Bouwes Bavinck J.N., Gorbalenya A.E., Feltkamp M.C. (2010). Discovery of a new human polyomavirus associated with trichodysplasia spinulosa in an immunocompromized patient. PLoS Pathog..

[12] Scuda N., Hofmann J., Calvignac-Spencer S., Ruprecht K., Liman P., Kühn J., Hengel H., Ehlers B. (2011). A novel human polyomavirus closely related to the african green monkey-derived lymphotropic polyomavirus. J. Virol..

[13] Buck C.B., Phan G.Q., Raiji M.T., Murphy P.M., McDermott D.H., McBride A.A. (2012). Complete genome sequence of a tenth human polyomavirus. J. Virol..

[14] Lim E.S., Reyes A., Antonio M., Saha D., Ikumapayi U.N., Adeyemi M., Stine O.C., Skelton R., Brennan D.C., Mkakosya R.S. (2013). Discovery of STL polyomavirus, a polyomavirus of ancestral recombinant origin that encodes a unique T antigen by alternative splicing. Virology.

[15] Korup S., Rietscher J., Calvignac-Spencer S., Trusch F., Hofmann J., Moens U., Sauer I., Voigt S., Schmuck R., Ehlers B. (2013). Identification of a novel human polyomavirus in organs of the gastrointestinal tract. PLoS ONE.

[16] Mishra N., Pereira M., Rhodes R.H., An P., Pipas J.M., Jain K., Kapoor A., Briese T., Faust P.L., Lipkin W.I. (2014). Identification of a novel polyomavirus in a pancreatic transplant recipient with retinal blindness and vasculitic myopathy. J. Infect. Dis..

[17] Gheit T., Dutta S., Oliver J., Robitaille A., Hampras S., Combes J.D., McKay-Chopin S., Le Calvez-Kelm F., Fenske N., Cherpelis B. (2017). Isolation and characterization of a novel putative human polyomavirus. Virology.

[18] Ondov B.D., Starrett G.J., Sappington A., Kostic A., Koren S., Buck C.B., Phillippy A.M. (2019). Mash Screen: High-throughput sequence containment estimation for genome discovery. Genome Biol..

[19] Calvignac-Spencer S., Feltkamp M.C., Daugherty M.D., Moens U., Ramqvist T., Johne R., Ehlers B. (2016). A taxonomy update for the family Polyomaviridae. Arch. Virol..

[20] Moens U., Calvignac-Spencer S., Lauber C., Ramqvist T., Feltkamp M.C.W., Daugherty M.D., Verschoor E.J., Ehlers B. (2017). ICTV Report Consortium. ICTV Virus Taxonomy Profile: Polyomaviridae. J. Gen. Virol..

[21] Kamminga S., van der Meijden E., Feltkamp M.C.W., Zaaijer H.L. (2018). Seroprevalence of fourteen human polyomaviruses determined in blood donors. PLoS ONE.

[22] Kourieh A., Combes J.D., Tommasino M., Dalstein V., Clifford G.M., Lacau St Guily J., Clavel C., Franceschi S., Gheit T. (2018). For The Split Study, G. Prevalence and risk factors of human polyomavirus infections in non-malignant tonsils and gargles: The SPLIT study. J. Gen. Virol..

[23] Wang Y., Keinonen A., Koskenmies S., Pitkänen S., Fyhrquist N., Sadeghi M., Mäkisalo H., Söderlund-Venermo M., Hedman K. (2019). Occurrence of newly discovered human polyomaviruses in skin of liver transplant recipients and their relation with squamous cell carcinoma in situ and actinic keratosis—a single-center cohort study. Transpl. Int..

[24] Fahsbender E., Altan E., Estrada M., Seguin M.A., Young P., Leutenegger C.M., Delwart E. (2019). Lyon-IARC Polyomavirus DNA in Feces of Diarrheic Cats. Microbiol. Resour. Announc..

[25] Gaboriaud P., Ferté M., Arnold F., Leblond V., Nicol J., Debare H., Le Meur M., Martini F., Tognon M., Touzé A. (2018). Age-specific seroprevalence of human polyomavirus 12 and Saint Louis and New Jersey polyomaviruses. Emerg. Microbes Infect..

[26] Bialasiewicz S., Rockett R.J., Barraclough K.A., Leary D., Dudley K.J., Isbel N.M., Sloots T.P. (2016). Detection of Recently Discovered Human Polyomaviruses in a Longitudinal Kidney Transplant Cohort. Am. J. Transpl..

[27] Li K., Zhang C., Zhao R., Xue Y., Yang J., Peng J., Jin Q. (2015). The prevalence of STL polyomavirus in stool samples from Chinese children. J. Clin..

[28] Herberhold S., Hellmich M., Panning M., Bartok E., Silling S., Akgül B., Wieland U. (2017). Human polyomavirus and human papillomavirus prevalence and viral load in non-malignant tonsillar tissue and tonsillar carcinoma. Med. Microbiol. Immunol..

[29] Bergallo M., Daprà V., Fava P., Ponti R., Calvi C., Montanari P., Novelli M., Quaglino P., Galliano I., Fierro M.T. (2018). DNA from Human Polyomaviruses, MWPyV, HPyV6, HPyV7, HPyV9 and HPyV12 in Cutaneous T-cell Lymphomas. Anticancer Res..

[30] Daprà V., Galliano I., Rassu M., Calvi C., Montanari P., Merlino C., Bergallo M. (2020). Lack of detection of HPyV12 DNA using real-time PCR in Italian infants with diarrhea. Minerva Pediatr..

[31] Kamminga S., van der Meijden E., Wunderink H.F., Touzé A., Zaaijer H.L., Feltkamp M.C.W. (2018). Development and Evaluation of a Broad Bead-Based Multiplex Immunoassay To Measure IgG Seroreactivity against Human Polyomaviruses. J. Clin. Microbiol..

[32] Gedvilaite A., Tryland M., Ulrich R.G., Schneider J., Kurmauskaite V., Moens U., Preugschas H., Calvignac-Spencer S., Ehlers B. (2017). Novel polyomaviruses in shrews (Soricidae) with close similarity to human polyomavirus 12. J. Gen. Virol..

[33] Schowalter R.M., Buck C.B. (2013). The Merkel cell polyomavirus minor capsid protein. PLoS Pathog..

[34] Madeira F., Park Y.M., Lee J., Buso N., Gur T., Madhusoodanan N., Basutkar P., Tivey A.R.N., Potter S.C., Finn R.D. (2019). The EMBL-EBI search and sequence analysis tools APIs in 2019. Nucleic Acids Res..

[35] Fanning E., Zhao K. (2009). SV40 DNA replication: From the A gene to a nanomachine. Virology.

[36] Kelly T.J. (1988). SV40 DNA replication. J. Biol. Chem..

[37] Farmerie W.G., Folk W.R. (1984). Regulation of polyomavirus transcription by large tumor antigen. Proc. Natl. Acad. Sci. USA.

[38] Tjian R. (1981). Regulation of viral transcription and DNA replication by the SV40 large T antigen. Curr. Top Microbiol. Immunol..

[39] Zenke M., Grundström T., Matthes H., Wintzerith M., Schatz C., Wildeman A., Chambon P. (1986). Multiple sequence motifs are involved in SV40 enhancer function. EMBO J..

[40] Rio D.C., Tjian R. (1984). Multiple control elements involved in the initiation of SV40 late transcription. J. Mol. Appl. Gen..

[41] Cowie A., Kamen R. (1984). Multiple binding sites for polyomavirus large T antigen within regulatory sequences of polyomavirus DNA. J. Virol..

[42] Jones K.A., Tjian R. (1984). Essential contact residues within SV40 large T antigen binding sites I and II identified by alkylation-interference. Cell.

[43] Lednicky J.A., Butel J.S. (2001). Simian virus 40 regulatory region structural diversity and the association of viral archetypal regulatory regions with human brain tumors. Semin. Cancer Biol..

[44] O’Neill F.J., Greenlee J.E., Carney H. (2003). The archetype enhancer of simian virus 40 DNA is duplicated during virus growth in human cells and rhesus monkey kidney cells but not in green monkey kidney cells. Virology.

[45] Lednicky J.A., Wong C., Butel J.S. (1995). Artificial modification of the viral regulatory region improves tissue culture growth of SV40 strain 776. Virus Res..

[46] Sroller V., Vilchez R.A., Stewart A.R., Wong C., Butel J.S. (2008). Influence of the viral regulatory region on tumor induction by simian virus 40 in hamsters. J. Virol..

[47] Muller W.J., Mueller C.R., Mes A.M., Hassell J.A. (1983). Polyomavirus origin for DNA replication comprises multiple genetic elements. J. Virol..

[48] Pomerantz B.J., Hassell J.A. (1984). Polyomavirus and simian virus 40 large T antigens bind to common DNA sequences. J. Virol..

[49] Herbomel P., Bourachot B., Yaniv M. (1984). Two distinct enhancers with different cell specificities coexist in the regulatory region of polyoma. Cell.

[50] Iacoangeli A., Melucci-Vigo G., Risuleo G., Santi E. (1995). Role of mouse polyomavirus late region in the control of viral DNA replication: A review. Biochimie.

[51] Jat P., Novak U., Cowie A., Tyndall C., Kamen R. (1982). DNA sequences required for specific and efficient initiation of transcription at the polyoma virus early promoter. Mol. Cell Biol..

[52] Mueller C.R., Mes-Masson A.M., Bouvier M., Hassell J.A. (1984). Location of sequences in polyomavirus DNA that are required for early gene expression in vivo and in vitro. Mol. Cell Biol..

[53] Rochford R., Campbell B.A., Villarreal L.P. (1990). Genetic analysis of the enhancer requirements for polyomavirus DNA replication in mice. J. Virol..

[54] Sekikawa K., Levine A.J. (1981). Isolation and characterization of polyoma host range mutants that replicate in nullipotential embryonal carcinoma cells. Proc. Natl. Acad. Sci. USA.

[55] Veldman G.M., Lupton S., Kamen R. (1985). Polyomavirus enhancer contains multiple redundant sequence elements that activate both DNA replication and gene expression. Mol. Cell Biol..

[56] White M.K., Khalili K. (2011). Pathogenesis of progressive multifocal leukoencephalopathy—revisited. J. Infect. Dis..

[57] Assetta B., Atwood W.J. (2017). The biology of JC polyomavirus. Biol. Chem..

[58] Chen N.N., Khalili K. (1995). Transcriptional regulation of human JC polyomavirus promoters by cellular proteins YB-1 and Pur alpha in glial cells. J. Virol..

[59] Sadowska B., Barrucco R., Khalili K., Safak M. (2003). Regulation of human polyomavirus JC virus gene transcription by AP-1 in glial cells. J. Virol..

[60] Romagnoli L., Sariyer I.K., Tung J., Feliciano M., Sawaya B.E., Del Valle L., Ferrante P., Khalili K., Safak M., White M.K. (2008). Early growth response-1 protein is induced by JC virus infection and binds and regulates the JC virus promoter. Virology.

[61] Pietropaolo V., Prezioso C., Bagnato F., Antonelli G. (2018). John Cunningham virus: An overview on biology and disease of the etiological agent of the progressive multifocal leukoencephalopathy. New Microbiol..

[62] Van Loy T., Thys K., Tritsmans L., Stuyver L.J. (2013). Quasispecies analysis of JC virus DNA present in urine of healthy subjects. PLoS ONE.

[63] Kenney S., Natarajan V., Strike D., Khoury G., Salzman N.P. (1984). JC virus enhancer-promoter active in human brain cells. Science.

[64] Yogo Y., Kitamura T., Sugimoto C., Ueki T., Aso Y., Hara K., Taguchi F. (1990). Isolation of a possible archetypal JC virus DNA sequence from nonimmunocompromised individuals. J. Virol..

[65] L’Honneur A.S., Leh H., Laurent-Tchenio F., Hazan U., Rozenberg F., Bury-Moné S. (2018). Exploring the role of NCCR variation on JC polyomavirus expression from dual reporter minicircles. PLoS ONE.

[66] Agostini H.T., Ryschkewitsch C.F., Stoner G.L. (1998). Rearrangements of archetypal regulatory regions in JC virus genomes from urine. Res. Virol..

[67] Bofill-Mas S., Clemente-Casares P., Major E.O., Curfman B., Girones R. (2003). Analysis of the excreted JC virus strains and their potential oral transmission. J. Neurovirol..

[68] Frisque R.J., Bream G.L., Cannella M.T. (1984). Human polyomavirus JC virus genome. J. Virol..

[69] Markowitz R.B., Dynan W.S. (1988). Binding of cellular proteins to the regulatory region of BK virus DNA. J. Virol..

[70] Moens U., Van Ghelue M. (2005). Polymorphism in the genome of non-passaged human polyomavirus BK: Implications for cell tropism and the pathological role of the virus. Virology.

[71] Rubinstein R., Pare N., Harley E.H. (1987). Structure and function of the transcriptional control region of nonpassaged BK virus. J. Virol..

[72] Bethge T., Ajuh E., Hirsch H.H. (2016). Imperfect Symmetry of Sp1 and Core Promoter Sequences Regulates Early and Late Virus Gene Expression of the Bidirectional BK Polyomavirus Noncoding Control Region. J. Virol..

[73] Bethge T., Hachemi H.A., Manzetti J., Gosert R., Schaffner W., Hirsch H.H. (2015). Sp1 sites in the noncoding control region of BK polyomavirus are key regulators of bidirectional viral early and late gene expression. J. Virol..

[74] Helle F., Brochot E., Handala L., Martin E., Castelain S., Francois C., Duverlie G. (2017). Biology of the BKPyV: An Update. Viruses.

[75] Moens U., Sundsfjord A., Flaegstad T., Traavik T. (1990). BK virus early RNA transcripts in stably transformed cells: Enhanced levels induced by dibutyryl cyclic AMP, forskolin and 12-O-tetradecanoylphorbol-13-acetate treatment. J. Gen. Virol..

[76] Moens U., Subramaniam N., Johansen B., Johansen T., Traavik T. (1994). A steroid hormone response unit in the late leader of the noncoding control region of the human polyomavirus BK confers enhanced host cell permissivity. J. Virol..

[77] Gorrill T.S., Khalili K. (2005). Cooperative interaction of p65 and C/EBPbeta modulates transcription of BKV early promoter. Virology.

[78] Anselmo A., Prezioso C., Saccà F.A., Di Lella F.M., Palmieri G., Tisone G., Pietropaolo V., Ciotti M. (2019). Kidney graft failure induced by BKPyV replication despite a strong reduction of the immunosuppressive therapy. J. Med. Virol..

[79] Arthur R.R., Shah K.V., Baust S.J., Santos G.W., Saral R. (1986). Association of BK viruria with hemorrhagic cystitis in recipients of bone marrow transplants. N. Engl. J. Med..

[80] Rosen S., Harmon W., Krensky A.M., Edelson P.J., Padgett B.L., Grinnell B.W., Rubino M.J., Walker D.L. (1983). Tubulo-interstitial nephritis associated with polyomavirus (BK type) infection. N. Engl. J. Med..

[81] McIlroy D., Halary F., Bressollette-Bodin C. (2019). Intra-patient viral evolution in polyomavirus-related diseases. Philos. Trans. R. Soc. B Biol. Sci..

[82] Seif I., Khoury G., Dhar R. (1979). The genome of human papovavirus BKV. Cell.

[83] Yang J.F., You J. (2020). Regulation of Polyomavirus Transcription by Viral and Cellular Factors. Viruses.

[84] Olsen G.H., Hirsch H.H., Rinaldo C.H. (2009). Functional analysis of polyomavirus BK non-coding control region quasispecies from kidney transplant recipients. J. Med. Virol..

[85] Degener A.M., Pietropaolo V., Di Taranto C., Jin L., Ameglio F., Cordiali-Fei P., Trento E., Sinibaldi L., Orsi N. (1999). Identification of a new control region in the genome of the DDP strain of BK virus isolated from PBMC. J. Med. Virol..

[86] Pietropaolo V., Videtta M., Fioriti D., Mischitelli M., Arancio A., Orsi N., Degener A.M. (2003). Rearrangement patterns of JC virus noncoding control region from different biological samples. J. Neurovirol..

[87] Mischitelli M., Fioriti D., Videtta M., Degener A.M., Antinori A., Cinque P., Giordano A., Pietropaolo V. (2005). Investigation on the role of cell transcriptional factor Sp1 and HIV-1 TAT protein in PML onset or development. J. Cell Physiol..

[88] Ciardi M.R., Zingaropoli M.A., Iannetta M., Prezioso C., Perri V., Pasculli P., Lichtner M., D’Ettorre G., Altieri M., Conte A. (2020). JCPyV NCCR analysis in PML patients with different risk factors: Exploring common rearrangements as essential changes for neuropathogenesis. Virol. J..

[89] Prezioso C., Zingaropoli M.A., Iannetta M., Rodio D.M., Altieri M., Conte A., Vullo V., Ciardi M.R., Palamara A.T., Pietropaolo V. (2020). Which is the best PML risk stratification strategy in natalizumab-treated patients affected by multiple sclerosis?. Mult. Scler. Relat. Disord..

[90] Prezioso C., Scribano D., Bellizzi A., Anzivino E., Rodio D.M., Trancassini M., Palamara A.T., Pietropaolo V. (2017). Efficient propagation of archetype JC polyomavirus in COS-7 cells: Evaluation of rearrangements within the NCCR structural organization after transfection. Arch. Virol..

[91] Prezioso C., Scribano D., Rodio D.M., Ambrosi C., Trancassini M., Palamara A.T., Pietropaolo V. (2018). COS-7-based model: Methodological approach to study John Cunningham virus replication cycle. Virol. J..

[92] Jiang M., Abend J.R., Johnson S.F., Imperiale M.J. (2009). The role of polyomaviruses in human disease. Virology.

[93] Babakir-Mina M., Ciccozzi M., Perno C.F., Ciotti M. (2013). The human polyomaviruses KI and WU: Virological background and clinical implications. APMIS.

[94] Jartti T., Jartti L., Ruuskanen O., Söderlund-Venermo M. (2012). New respiratory viral infections. Curr. Opin. Pulm. Med..

[95] Gelfand Y., Rodriguez A., Benson G. (2007). TRDB—the Tandem Repeats Database. Nucleic Acids Res..

[96] Song X., Van Ghelue M., Ludvigsen M., Nordbø S.A., Ehlers B., Moens U. (2016). Characterization of the non-coding control region of polyomavirus KI isolated from nasopharyngeal samples from patients with respiratory symptoms or infection and from blood from healthy blood donors in Norway. J. Gen. Virol..

[97] Li K., Guo J., Zhao R., Xue Y., Chen L., Yang J., Peng J., Jin Q. (2013). Prevalence of 10 human polyomaviruses in fecal samples from children with acute gastroenteritis: A case-control study. J. Clin. Microbiol..

[98] Tsunoda T., Takagi T. (1999). Estimating transcription factor bindability on DNA. Bioinformatics.

[99] Jackstadt R., Röh S., Neumann J., Jung P., Hoffmann R., Horst D., Berens C., Bornkamm G.W., Kirchner T., Menssen A. (2013). AP4 is a mediator of epithelial-mesenchymal transition and metastasis in colorectal cancer. J. Exp. Med..

[100] Uhlén M., Fagerberg L., Hallstrom B.M., Lindskog C., Oksvold P., Mardinoglu A., Sivertsson Å., Kampf C., Sjöstedt E., Asplund A. (2015). Proteomics. Tissue-based map of the human proteome. Science.

[101] Csoma E., Lengyel G., Bányai K., Takács P., Ánosi N., Marton S., Mátyus M., Pászti E., Gergely L., Szűcs A. (2018). Study of Karolinska Institutet and Washington University polyomaviruses in tonsil, adenoid, throat swab and middle ear fluid samples. Future Microbiol..

[102] Moens U., Van Ghelue M., Ludvigsen M., Korup-Schulz S., Ehlers B. (2015). Early and late promoters of BKPyV, MCPyV, TSPyV, and HPyV12 are among the strongest of all known human polyomaviruses in 10 different cell lines. J. Gen. Virol..

[103] Sood R., Kamikubo Y., Liu P. (2017). Role of RUNX1 in hematological malignancies. Blood.

[104] Gosert R., Rinaldo C.H., Funk G.A., Egli A., Ramos E., Drachenberg C.B., Hirsch H.H. (2008). Polyomavirus BK with rearranged noncoding control region emerge in vivo in renal transplant patients and increase viral replication and cytopathology. J. Exp. Med..

[105] Gosert R., Kardas P., Major E.O., Hirsch H.H. (2010). Rearranged JC virus noncoding control regions found in progressive multifocal leukoencephalopathy patient samples increase virus early gene expression and replication rate. J. Virol..

[106] Feng H., Kwun H.J., Liu X., Gjoerup O., Stolz D.B., Chang Y., Moore P.S. (2011). Cellular and viral factors regulating Merkel cell polyomavirus replication. PLoS ONE.

[107] Kwun H.J., Guastafierro A., Shuda M., Meinke G., Bohm A., Moore P.S., Chang Y. (2009). The minimum replication origin of merkel cell polyomavirus has a unique large T-antigen loading architecture and requires small T-antigen expression for optimal replication. J. Virol..

[108] Hashida Y., Higuchi T., Matsui K., Shibata Y., Nakajima K., Sano S., Daibata M. (2018). Genetic Variability of the Noncoding Control Region of Cutaneous Merkel Cell Polyomavirus: Identification of Geographically Related Genotypes. J. Infect. Dis..

[109] Delbue S., Franciotta D., Giannella S., Dolci M., Signorini L., Ticozzi R., D’Alessandro S., Campisciano G., Comar M., Ferrante P. (2019). Human Polyomaviruses in the Cerebrospinal Fluid of Neurological Patients. Microorganisms.

[110] Prezioso C., Obregon F., Ambroselli D., Petrolo S., Checconi P., Rodio D.M., Coppola L., Nardi A., Vito C., Sarmati L. (2020). Merkel Cell Polyomavirus (MCPyV) in the Context of Immunosuppression: Genetic Analysis of Noncoding Control Region (NCCR) Variability among a HIV-1-Positive Population. Viruses.

[111] White M.K., Safak M., Khalili K. (2009). Regulation of gene expression in primate polyomaviruses. J. Virol..

[112] Ajuh E.T., Wu Z., Kraus E., Weissbach F.H., Bethge T., Gosert R., Fischer N., Hirsch H.H. (2018). Novel Human Polyomavirus Noncoding Control Regions Differ in Bidirectional Gene Expression according to Host Cell, Large T-Antigen Expression, and Clinically Occurring Rearrangements. J. Virol..

[113] Prezioso C., Ciotti M., Obregon F., Ambroselli D., Rodio D.M., Cudillo L., Gaziev J., Mele A., Nardi A., Favalli C. (2019). Polyomaviruses shedding in stool of patients with hematological disorders: Detection analysis and study of the non-coding control region’s genetic variability. Med. Microbiol. Immunol..

[114] Bialasiewicz S., Lambert S.B., Whiley D.M., Nissen M.D., Sloots T.P. (2009). Merkel cell polyomavirus DNA in respiratory specimens from children and adults. Emerg. Infect. Dis..

[115] Kantola K., Sadeghi M., Lahtinen A., Koskenvuo M., Aaltonen L.M., Möttönen M., Rahiala J., Saarinen-Pihkala U., Riikonen P., Jartti T. (2009). Merkel cell polyomavirus DNA in tumor-free tonsillar tissues and upper respiratory tract samples: Implications for respiratory transmission and latency. J. Clin. Virol..

[116] Babakir-Mina M., Ciccozzi M., Lo Presti A., Greco F., Perno C.F., Ciotti M. (2010). Identification of Merkel cell polyomavirus in the lower respiratory tract of Italian patients. J. Med. Virol..

[117] Abedi Kiasari B., Vallely P.J., Klapper P.E. (2011). Merkel cell polyomavirus DNA in immunocompetent and immunocompromised patients with respiratory disease. J. Med. Virol..

[118] Shikova E., Emin D., Alexandrova D., Shindov M., Kumanova A., Lekov A., Moens U. (2017). Detection of Merkel Cell Polyomavirus in Respiratory Tract Specimens. Intervirology.

[119] Iaria M., Caccuri F., Apostoli P., Giagulli C., Pelucchi F., Padoan R.F., Caruso A., Fiorentini S. (2015). Detection of KI WU and Merkel cell polyomavirus in respiratory tract of cystic fibrosis patients. Clin. Microbiol. Infect..

[120] Prezioso C., Di Lella F.M., Rodio D.M., Bitossi C., Trancassini M., Mele A., de Vito C., Antonelli G., Pietropaolo V. (2019). Merkel Cell Polyomavirus DNA Detection in Respiratory Samples: Study of a Cohort of Patients Affected by Cystic Fibrosis. Viruses.

[121] Abdulsalam I., Rasheed K., Sveinbjørnsson B., Ehlers B., Moens U. (2020). Promoter activity of Merkel cell Polyomavirus variants in human dermal fibroblasts and a Merkel cell carcinoma cell line. Virol. J..

[122] Shuda M., Feng H., Kwun H.J., Rosen S.T., Gjoerup O., Moore P.S., Chang Y. (2008). T antigen mutations are a human tumor-specific signature for Merkel cell polyomavirus. Proc. Natl. Acad. Sci. USA.

[123] Hashida Y., Higuchi T., Matsuzaki S., Nakajima K., Sano S., Daibata M. (2018). Prevalence and Genetic Variability of Human Polyomaviruses 6 and 7 in Healthy Skin Among Asymptomatic Individuals. J. Infect. Dis..

[124] Ho J., Jedrych J.J., Feng H., Natalie A.A., Grandinetti L., Mirvish E., Crespo M.M., Yadav D., Fasanella K.E., Proksell S. (2015). Human polyomavirus 7-associated pruritic rash and viremia in transplant recipients. J. Infect. Dis..

[125] Nguyen K.D., Lee E.E., Yue Y., Stork J., Pock L., North J.P., Vandergriff T., Cockerell C., Hosler G.A., Pastrana D.V. (2017). Human polyomavirus 6 and 7 are associated with pruritic and dyskeratotic dermatoses. J. Am. Acad. Derm..

[126] Canavan T.N., Baddley J.W., Pavlidakey P., Tallaj J.A., Elewski B.E. (2018). Human polyomavirus-7-associated eruption successfully treated with acitretin. Am. J. Transpl..

[127] Smith S.D.B., Erdag G., Cuda J.D., Rangwala S., Girardi N., Bibee K., Orens J.B., Prono M.D., Toptan T., Loss M.J. (2018). Treatment of human polyomavirus-7-associated rash and pruritus with topical cidofovir in a lung transplant patient: Case report and literature review. Transpl. Infect. Dis..

[128] Rosenstein R.K., Pastrana D.V., Starrett G.J., Sapio M.R., Hill N.T., Jo J.H., Lee C.R., Iadarola M.J., Buck C.B., Kong H.H. (2020). Host-Pathogen Interactions in Human Polyomavirus 7 (HPyV7)-associated Pruritic Skin Eruption. J. Investig. Derm..

[129] Klufah F., Mobaraki G., Chteinberg E., Alharbi R.A., Winnepenninckx V., Speel E.J.M., Rennspiess D., Olde Damink S.W., Neumann U.P., Kurz A.K. (2020). High Prevalence of Human Polyomavirus 7 in Cholangiocarcinomas and Adjacent Peritumoral Hepatocytes: Preliminary Findings. Microorganisms.

[130] Schrama D., Groesser L., Ugurel S., Hafner C., Pastrana D.V., Buck C.B., Cerroni L., Theiler A., Becker J.C. (2014). Presence of human polyomavirus 6 in mutation-specific BRAF inhibitor-induced epithelial proliferations. JAMA Dermatol..

[131] Hampras S.S., Locke F.L., Chavez J.C., Patel N.S., Giuliano A.R., Miller K., Gheit T., Tommasino M., Rollison D.E. (2018). Prevalence of cutaneous viral infections in incident cutaneous squamous cell carcinoma detected among chronic lymphocytic leukemia and hematopoietic stem cell transplant patients. Leuk. Lymphoma.

[132] Kamminga S., van der Meijden E., de Brouwer C., Feltkamp M., Zaaijer H. (2019). Prevalence of DNA of fourteen human polyomaviruses determined in blood donors. Transfusion.

[133] Hashida Y., Higuchi T., Tanaka M., Shibata Y., Nakajima K., Sano S., Daibata M. (2019). Prevalence and Viral Loads of Cutaneous Human Polyomaviruses in the Skin of Patients With Chronic Inflammatory Skin Diseases. J. Infect. Dis..

[134] Harrison C., Jiang T., Banerjee P., Meinke G., D’Abramo C.M., Schaffhausen B., Bohm A. (2013). Polyomavirus large T antigen binds symmetrical repeats at the viral origin in an asymmetrical manner. J. Virol..

[135] Toptan T., Yousem S.A., Ho J., Matsushima Y., Stabile L.P., Fernández-Figueras M.T., Bhargava R., Ryo A., Moore P.S., Chang Y. (2016). Survey for human polyomaviruses in cancer. JCI Insight.

[136] Kazem S., Lauber C., van der Meijden E., Kooijman S., Kravchenko A.A., Feltkamp M.C., Gorbalenya A.E. (2016). Limited variation during circulation of a polyomavirus in the human population involves the COCO-VA toggling site of Middle and Alternative T-antigen(s). Virology.

[137] Sauvage V., Foulongne V., Cheval J., Ar Gouilh M., Pariente K., Dereure O., Manuguerra J.C., Richardson J., Lecuit M., Burguière A. (2011). Human polyomavirus related to African green monkey lymphotropic polyomavirus. Emerg. Infect. Dis..

[138] Lednicky J.A., Butel J.S., Luetke M.C., Loeb J.C. (2014). Complete genomic sequence of a new Human polyomavirus 9 strain with an altered noncoding control region. Virus Genes..

[139] Moens U., Song X., Van Ghelue M., Lednicky J.A., Ehlers B. (2017). A Role of Sp1 Binding Motifs in Basal and Large T-Antigen-Induced Promoter Activities of Human Polyomavirus HPyV9 and Its Variant UF-1. Int. J. Mol. Sci..

[140] Siebrasse E.A., Reyes A., Lim E.S., Zhao G., Mkakosya R.S., Manary M.J., Gordon J.I., Wang D. (2012). Identification of MW polyomavirus, a novel polyomavirus in human stool. J. Virol..

[141] Rockett R.J., Sloots T.P., Bowes S., O’Neill N., Ye S., Robson J., Whiley D.M., Lambert S.B., Wang D., Nissen M.D. (2013). Detection of novel polyomaviruses, TSPyV, HPyV6, HPyV7, HPyV9 and MWPyV in feces, urine, blood, respiratory swabs and cerebrospinal fluid. PLoS ONE.

[142] Pastrana D.V., Fitzgerald P.C., Phan G.Q., Raiji M.T., Murphy P.M., McDermott D.H., Velez D., Bliskovsky V., McBride A.A., Buck C.B. (2013). A divergent variant of the eleventh human polyomavirus species, saint louis polyomavirus. Genome Announc..

[143] Peng J., Li K., Zhang C., Jin Q. (2016). MW polyomavirus and STL polyomavirus present in tonsillar tissues from children with chronic tonsillar disease. Clin. Microbiol. Infect..

[144] Liu W., Yang R., Payne A.S., Schowalter R.M., Spurgeon M.E., Lambert P.F., Xu X., Buck C.B., You J. (2016). Identifying the Target Cells and Mechanisms of Merkel Cell Polyomavirus Infection. Cell Host Microbe.

[145] Pietropaolo V., Prezioso C., Moens U. (2020). Merkel Cell Polyomavirus and Merkel Cell Carcinoma. Cancers.

[146] Kazem S., van der Meijden E., Feltkamp M.C. (2013). The trichodysplasia spinulosa-associated polyomavirus: Virological background and clinical implications. APMIS.

[147] Narayanan D., Rady P.L., Tyring S.K. (2020). Recent developments in trichodysplasia spinulosa disease. Transpl. Infect. Dis..

